# The fungal-specific β-glucan-binding lectin FGB1 alters cell-wall composition and suppresses glucan-triggered immunity in plants

**DOI:** 10.1038/ncomms13188

**Published:** 2016-10-27

**Authors:** Stephan Wawra, Philipp Fesel, Heidi Widmer, Malte Timm, Jürgen Seibel, Lisa Leson, Leona Kesseler, Robin Nostadt, Magdalena Hilbert, Gregor Langen, Alga Zuccaro

**Affiliations:** 1 University of Cologne, Botanical Institute, Cluster of Excellence on Plant Sciences (CEPLAS), 50674 Cologne, Germany; 2 University of Würzburg, Institute of Organic Chemistry, 97074 Würzburg, Germany; 3 Max-Planck-Institute for Terrestrial Microbiology, Department of Organismic Interactions, 35043 Marburg, Germany

## Abstract

β-glucans are well-known modulators of the immune system in mammals but little is known about β-glucan triggered immunity *in planta*. Here we show by isothermal titration calorimetry, circular dichroism spectroscopy and nuclear magnetic resonance spectroscopy that the *FGB1* gene from the root endophyte *Piriformospora indica* encodes for a secreted fungal-specific β-glucan-binding lectin with dual function. This lectin has the potential to both alter fungal cell wall composition and properties, and to efficiently suppress β-glucan-triggered immunity in different plant hosts, such as *Arabidopsis*, barley and *Nicotiana benthamiana*. Our results hint at the existence of fungal effectors that deregulate innate sensing of β-glucan in plants.

## Abstract (web summary)

β-glucans derived from fungal cell walls can trigger immune responses in animals, yet their roles in plant-fungal interactions are less well known. Here, Wawra *et al*. show that the FGB1 proteins, secreted by the fungal endophyte *P. indica*, can alter fungal cell wall composition and suppress immune responses in plants.

The ability to distinguish self from non-self is a fundamental aspect of any immune system. Plants rely on the innate ability of each cell to recognize invaders and are capable of perceiving and responding to pathogenic or beneficial microbes via a multilayered, highly specific and effective immune system comprising surveillance of non-self, damaged-self and altered-self as danger signals^1^,^2^. To protect and defend against microbial invaders, but also to start the symbiotic program, plants need to detect the presence of microbes by means of pattern recognition receptors that respond to specific microbe-associated molecular patterns (MAMPs)^2^,^3^. MAMPs are usually slowly evolving components of microbes with crucial biological functions that are not present in the host^4^. During plant colonization, the first microbial-derived structure that makes physical contact with the host cell is the cell wall. In pathogenic as well as in beneficial fungi an important structural building block of the cell wall is chitin, a well-known elicitor of immune responses in plants^5^. Over the past 10 years, several plant receptors for chitin have been identified and characterized as have different microbial strategies to evade chitin recognition, including modification of the cell wall carbohydrate composition and secretion of effectors^6^,^7^,^8^. In spite of its strong elicitor activity, chitin represents only a small percentage of the fungal cell wall. By far the most abundant cell wall polysaccharide in the majority of fungi is β-glucan, a well characterized elicitor in fungus-animal systems^9^. Most β-glucans originating from the fungal cell wall are made of glucosyl units joined by β-1,3-linkages often branched via β-1,6-linked glucose residues^10^. Whereas β-1,3-glucan is found also in the cell wall of plants, β-1,6-glucan is a cell wall constituent specific to fungi and to members of the Stramenopiles, thereby representing a potential MAMP. Despite its abundance, studies focusing on the possible role of β-glucan as a MAMP are rare and the mechanisms of β-glucan perception and signalling in plants are largely unknown^10^. Most plant hosts secrete a large plethora of different hydrolytic enzymes. These include chitinases and glucanases that target fungal cell wall constituents to impact cell wall integrity and to release MAMPs. Direct mechanisms to evade β-glucan recognition by plant-associated fungi have not yet been reported but considering the widespread presence of β-glucan at the fungal cell wall, it is conceivable that plant-associated fungi have evolved sophisticated mechanisms to protect their glucan fibrils from hydrolysis and detection.

The root endophyte *Piriformospora indica* (Basidiomycota, Sebacinales) is a fungal symbiont that colonizes inter- and intracellularly the root epidermal and cortex cells of a broad range of unrelated plants, including the monocotyledonous cereal crop *Hordeum vulgare* (barley) and the dicotyledonous plant *Arabidopsis thaliana*, with a biphasic lifestyle. The initial biotrophic phase is followed by a stage where the fungus is more often found in dead or dying host cells secreting a large variety of hydrolytic enzymes that degrade plant cell walls and proteins. This is especially evident in barley where the symbiont rapidly undergoes a nutritional switch to saprotrophy that correlates with nitrogen limitation and is associated with the production of thinner hyphae in dead root cells^11^.

Here we report on the identification and functional characterization of FGB1 (Fungal Glucan-Binding 1, PIIN_03211), a small fungal-specific protein from the root endophyte *P. indica* induced during colonization of barley and *Arabidopsis*. By cytological and biochemical approaches, we show that FGB1 is a secreted lectin that binds β-glucan, alters fungal cell wall composition and suppresses glucan triggered reactive oxygen species (ROS) production in different plants, effecting fungal colonization.

## Results

### FGB1 is a fungal-specific lectin

In order to identify possible compatibility factors in the interaction of *P. indica* with different hosts, we recently performed global characterization of fungal transcriptional responses to barley and *Arabidopsis* at different developmental stages^11^,^12^. The majority of induced genes encoding small secreted proteins (SSPs; <300 amino acids), which are possibly enriched in effectors^13^, were either barley or *Arabidopsis* responsive, suggesting that colonization of different hosts might require exploitation of host dependently induced effectors that interact with elements characteristic to each host^11^,^14^. A small set of *P. indica* genes encoding SSPs, however, was expressed in both hosts at comparable symbiotic stages and hence may be enriched for general determinants of compatibility that target conserved recognition and signalling pathways in different hosts^11^.

**Figure 1:**
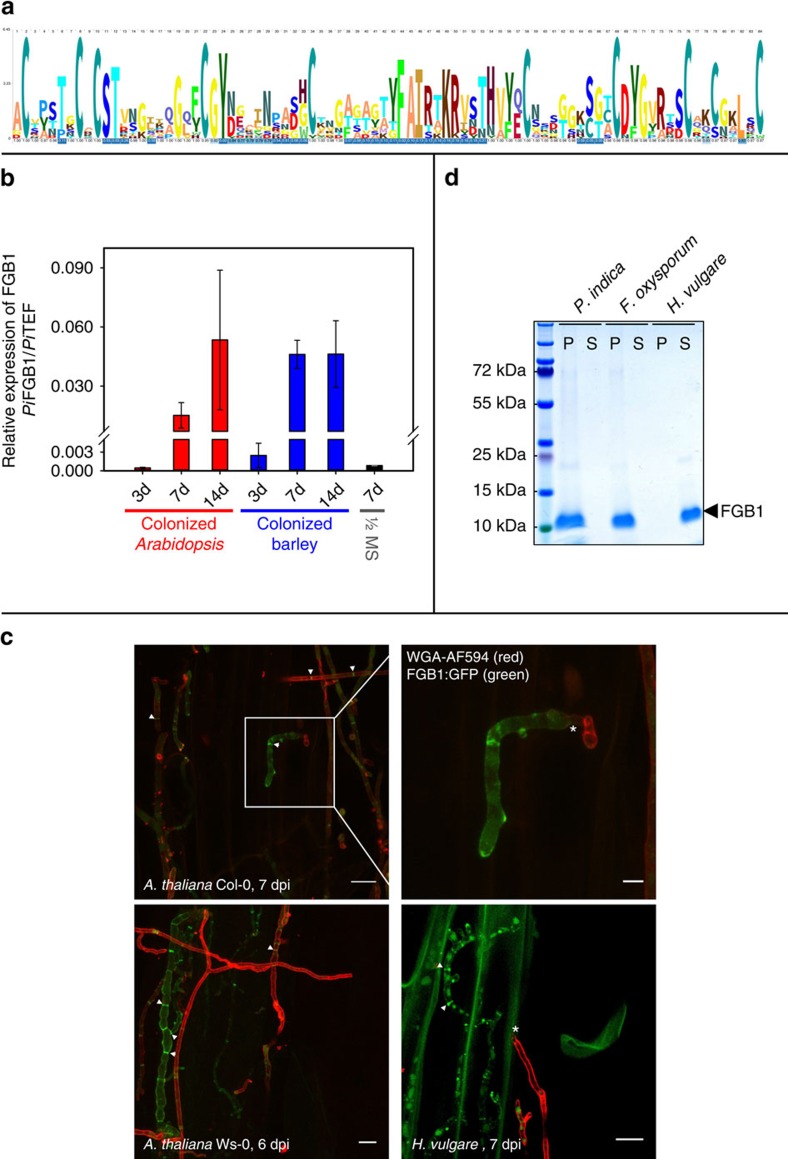
The small secreted *P. indica* protein FGB1 binds to fungal cell walls and harbours a fungal-specific lectin domain. (**a**) Protein sequence logo of FGB1 (PIIN_03211) obtained after alignment of the mature protein sequence with homologous sequences from different fungi. (**b**) *P. indica* FGB1 transcript levels in barley (*Hordeum vulgare*) and *Arabidopsis* Col-0 roots grown on ½ MS medium inoculated with spores and harvested 3, 7 and 14 dpi and on control ½ MS medium (7 dpi) relative to transcription elongation factor (TEF, PIIN_03008). Error bars are s.d.'s calculated from three biological replicate samples (eight technical replicates each). (**c**) FGB1:GFP localizes to the fungal septa (arrowheads) and cell wall in colonized *A. thaliana* Col-0 (7 dpi) and Ws-0 roots (6 dpi), as well as barley roots (7 dpi). The asterisks show the entry point of the hyphae into the root cell. The chitin dye Alexa Fluor 594 labelled wheat germ agglutinin is shown in red (see Supplementary Figs 1 and 3 for further details). Scale bars, 10 μm. Scale bar on the detail image (top right corner) is 3 μm. (**d**) SDS–PAGE showing the outcome of a pull down experiment probing the binding ability of FGB1 to protein free cell wall extracts of *P. indica*, *F. oxysporum* and barley roots. P, insoluble pellet fraction; S, supernatant. Incubation of FGB1 (20 μM) with the insoluble polysaccharides (10 mg each) was carried out for 1 h at room temperature in 25 mM sodium-acetate buffer (pH 5.0) containing 500 mM NaCl.

One of these putative *P. indica* core effectors is FGB1, a 104-amino-acid residue long protein. The amino acid sequence of FGB1 is similar to that of other uncharacterized proteins predicted from the genome sequences of fungi (Supplementary Fig. 1a). No homologous sequences have been found outside the fungal kingdom. Multiple alignment among these proteins shows that several cysteine residues are invariant, suggesting that these proteins may be capable of forming intramolecular disulfide bonds (Fig. 1a, Supplementary Fig. 1a). Interestingly, we found a homologue (*E*-value 2e-07) of *P. indica* FGB1 in the genome of the root pathogen *Fusarium oxysporum* (Ascomycota, Hypocreales). This gene named Pep2 was listed as an effector candidate because it is present in cotton wilt pathogenic strains of *F. oxysporum* f.sp. *vasinfectum* and absent in the non-pathogenic cotton field soil isolates, but was never functionally characterized^15^. Real-time PCR analyses of *P. indica* colonized roots of barley and *A. thaliana* on ½ MS confirmed that FGB1 is transcriptionally induced during colonization of both hosts compared with controls (½ MS without host plants) (Fig. 1b). FGB1 is not exclusively expressed *in planta*, but it represents the most abundant secreted protein in culture filtrate of *P. indica* grown in liquid complete medium (CM), whereas its production is suppressed in liquid yeast nitrogen base (YNB) medium (Supplementary Fig. 1b,c). The high abundance of FGB1 in CM culture filtrate allowed us to establish a purification strategy for the native FGB1 (Supplementary Fig. 1c). Liquid chromatography–mass spectrometry (MS)/MS analysis of the *P. indica* secreted cationic protein fraction at pH 7 (Supplementary Fig. 2a) and molecular weight determination (Supplementary Fig. 2b) showed that the native FGB1 is a non-glycosylated SSP of 6,219 Da stabilized by four disulfide bridges. Localization studies using *P. indica* strains expressing a FGB1:green fluorescent protein (GFP) reporter fusion construct under the control of the native promoter showed that FGB1 is present at the cell wall and septa of *P. indica* in colonized *Arabidopsis* (accessions Columbia, Col-0 and Wassilewskija, Ws-0) and barley (cultivar Golden Promise) roots and during axenic growth in liquid CM but not in YNB medium (Fig. 1c, Supplementary Fig. 3). We thus examined the affinity of this SSP to fungal and plant cell wall polymers using pull-down assays. The native FGB1 showed specific binding to fungal cell walls but not to barley cell wall components as it co-precipitated only with insoluble protein-free cell wall preparations of *P. indica* and *F. oxysporum* (Fig. 1d).

### FGB1 specifically binds β-1,6-linked glucose residues

**Figure 2:**
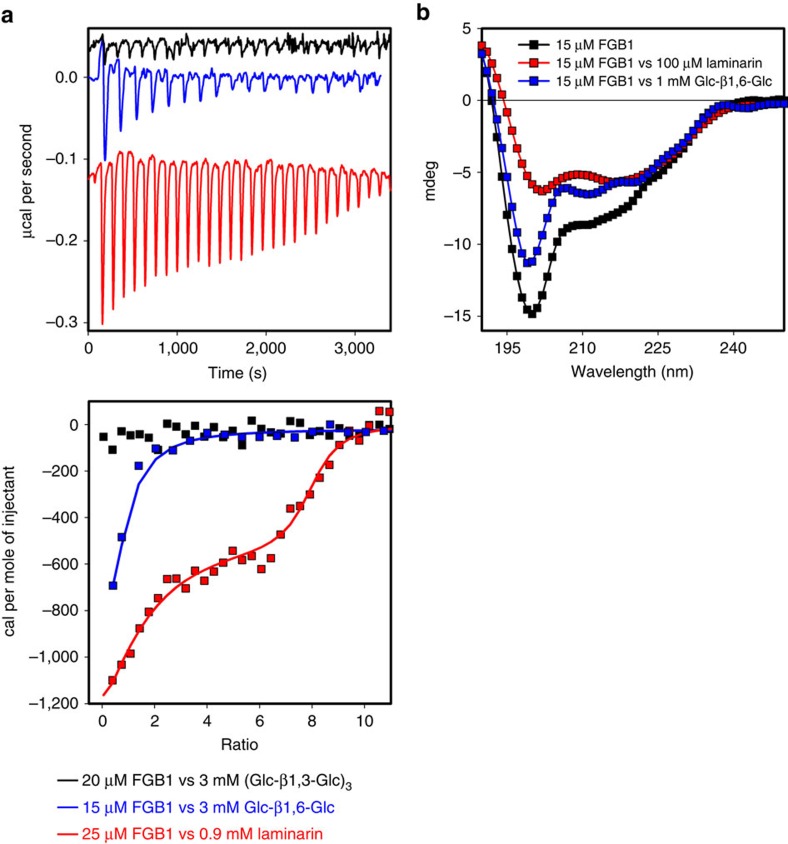
FGB1 specifically binds to β-glucan and to the disaccharide gentiobiose. (**a**) ITC profiles showing titrations of laminarihexaose [(Glc-β-1,3-Glc)_3_] (black line), gentiobiose (Glc-β-1,6-Glc) (blue line) and laminarin (red line) to FGB1. The laminarin titration was fitted using a model that allows binding to two equivalent and independent sites. Assuming a molecular mass of laminarin of 5 kDa the following parameters were obtained: *N*_1_=6.8±1.09, *K*_1_=2.14 × 10^6^±1.97 × 10^7^ M^−1^, Δ*H*_1_=−508±128 cal mol^−1^, Δ*S*_1_=27.2 cal mol^−1^ per degree and *N*_2_=1.02±0.9, *K*_2_=1.19 × 10^7^±6.11 × 10^7^ M^−1^, Δ*H*_2_=−1971±251 cal mol^−1^, Δ*S*_2_=25.7 cal mol^−1^ per degree. The titration of gentiobiose to FGB1 was fitted to a single-side-binding model (*N*=0.854±0.243, *K*=8.79 × 10^4^±6.88 × 10^5^ M^−1^, Δ*H*=−563.9±241 cal mol^−1^, Δ*S*=19.4 cal mol^−1^ per degree). No binding of FGB1 to laminarihexaose was observed. Concentrations of the stock solutions are indicated. All titrations were baseline corrected and substracted with the corresponding control titration of ligand into water. Errors correspond to the s.d. of the nonlinear least-squares fit of the data points of the titration curve. (**b**) The circular dichroism-spectrum of 15 μM FGB1 (black square) shows a significant shift in the secondary structure in the presence of 1 mM gentiobiose (blue square) or 100 μM laminarin (red square). Spectra were corrected with the spectra obtained for the individual ligands (see Supplementary Fig. 4 for further details).

To examine the specificity of FGB1 to different ligands, isothermal titration calorimetric (ITC) experiments were performed using soluble oligosaccharides and polymers present in fungal cell walls. The results showed that FGB1 binds with high affinity to laminarin (Sigma), a β-1,3-linked glucose polymer containing β-1,6-glycosidic side branches derived from brown algae (Stramenopiles) (Fig. 2a, Supplementary Fig. 4a). In addition, FGB1 was able to bind the chemically synthesized gentiobiose, a disaccharide composed of two units of D-glucose joined with a β-1,6 linkage but not β-1,3-laminarihexaose (Megazyme), α-glucan (chemically synthesized) and chitin oligosaccharides (Megazyme) (Fig. 2a, Supplementary Fig. 4a,b,c). The ITC measurements showed that FGB1 strongly interacts with laminarin in a dual-binding event. A first binding transition appeared at a protein:ligand ratio of 1:1 with a corresponding binding constant of ∼80 nM. A second transition occurred at a molecular ratio of 1:7 yielding a Kd value of about 800 nM (Fig. 2a, Supplementary Fig. 4a). Circular dichroism measurements revealed that binding to laminarin induces a conformational change in FGB1 that increases the proportion of α-helical structure (Fig. 2b). Binding to gentiobiose also induced a structural alteration, but this was less pronounced, suggesting that β-1,3-linkages may also contribute to the efficient binding of FGB1 to laminarin (Fig. 2b). Furthermore, circular dichroism measurements of recombinant (His)_6_FGB1^19–104^ produced in *E. coli* revealed that correct disulfide bridge formation is crucial for the proper function of this protein (Supplementary Fig. 4d). Therefore, we concluded that FGB1 is a novel type of disulfide-bridge-containing lectin that specifically binds β-glucan via β-1,6-linked glucose moieties in a context of a β-1,3 glucan backbone. The questions arose whether and to what extent binding of FGB1 to β-glucan at the fungal cell wall may alter wall stress responses and/or composition. In order to answer these questions, we attempted to express FGB1:GFP under the control of the constitutive *Pi*GPD promoter in *P. indica*. No strains constitutively expressing significant amounts of FGB1:GFP were retrieved (Supplementary Fig. 5). To test the functionality of this construct we expressed it in the smut pathogenic fungus *Ustilago maydis* (Basidiomycota, Ustilaginales) that lacks a FGB1 homologue. Expression of the ^Prom*Pi*GPD^FGB1:GFP fusion in *U. maydis* led to the accumulation of specific GFP signals at the cell wall indicating that the construct was functional (Supplementary Fig. 6a). These observations support findings from other fungal systems showing that overexpression of endogenous genes driven by strong constitutive promoters may lead to silencing of both, the extra gene copy and the endogenous gene^16^.

### FGB1 alters fungal cell wall proprieties and composition

**Figure 3:**
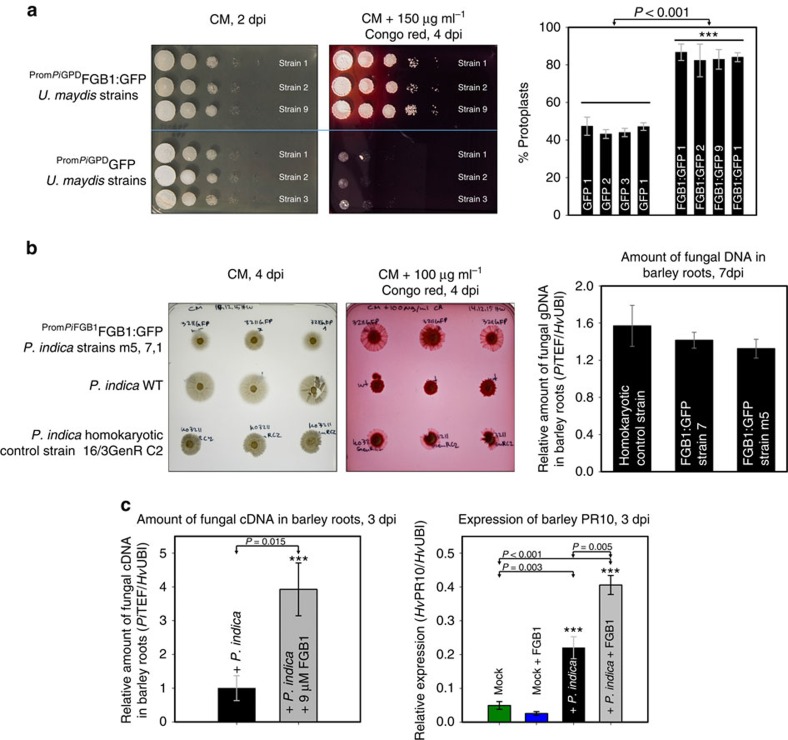
FGB1 confers resistance to Congo red-mediated cell wall stress and increases plant colonization by fungi. (**a**) Drop dilution series of *U. maydis* SG200 strains expressing either GFP or FGB1:GFP. Growth of three independent strains were tested on complete medium (CM) and CM supplemented with 150 μg ml^−1^ CR. *U. maydis* FGB1:GFP strains show an increased sensitivity towards chitinase activity (*Trichoderma viride*) compared with GFP expressing control strains. Protoplastation was carried out in 20 mM sodium citrate containing 1 M sorbitol, pH 5.8 for 15 min at 37 °C using 20 mg ml^−1^ chitinase. Data were obtained from four independent biological replicates carried out in three technical replicates using three independent strains by counting protoplasts and non-protoplasted cells. Error bars show s.d. Significance was calculated using the paired *t*-test algorithm of SigmaPlot. (**b**) To test if expression of ^Prom*Pi*FGB1^FGB1:GFP in *P. indica* also increases resistance to CR, plugs of identical size from the active growth zone of 7-day old culture plates were transferred onto CM with and without CR. Growth of *P. indica*^Prom*Pi*FGB1^FGB1:GFP strains was compared with that of the homokaryotic control strain and the dikaryotic WT strain. *P. indica*^Prom*Pi*FGB1^FGB1:GFP strains are more resistant to CR compared with the controls. At least four biological repetitions were done. No significant differences in barley colonization by *P. indica*^Prom*Pi*FGB1^FGB1:GFP strains compared with the homokaryotic control strain was observed at 7 dpi (see Supplementary Figs 6–8 for further details). Bars show the average of five independent biological experiments each done with two technical replicates. Error bars show the s.d. (**c**) Left: addition of 9 μM native FGB1 leads to a ∼4 fold increased *P. indica* colonization of barley roots at 3 dpi. Error bars show the standard error of the mean from eight independent biological replicates performed with three independently purified FGB1 batches. Right: Relative expression levels of barley PR10, a PR gene significantly induced during root colonization by *P. indica*^24^. Asterisks indicate significant difference calculated using the paired *t*-test algorithm of SigmaPlot.

To test for altered cell wall susceptibility, *U. maydis* strains expressing FGB1:GFP and control strains expressing GFP were cultured in the presence of Calcofluor white (CFW), Congo red (CR) or H_2_O_2_, three chemicals that are commonly used to identify cell wall mutants in fungi. CFW and CR are reported to interact *in vitro* with various β-linked glucans and *in vivo* with nascent chitin chains thereby inhibiting cell wall assembly^17^. Whereas CFW preferentially interacts with chitin^17^, CR is speculated to additionally interfere with β-glucan synthesis^18^. Expression of FGB1 in *U. maydis* did not alter the speed of growth in CM or the morphology of fungal colonies but increased resistance to H_2_O_2_ and in particular to CR but not to CFW (Fig. 3a, Supplementary Fig. 6b). To test if the FGB1 overexpression phenotype in *U. maydis* could be reproduced in *P. indica*, we assessed the growth of the ^Prom*Pi*FGB1^FGB1:GFP *P. indica* strains on CM plates or CM plates containing CR. On CM plates the ^Prom*Pi*FGB1^FGB1:GFP *P. indica* strains and the control strains carrying a gene for resistance to geneticin displayed a slower growth compared with the WT. This is maybe due to the fact that the FGB1:GFP and the control strains obtained from protoplast-mediated transformation were homokaryotic whereas the WT strain is dikaryotic (Supplementary Fig. 7). In our hands, the protoplast-mediated transformation led to regeneration of *P. indica* homokaryotic strains in 40 to 85% of all cases (Supplementary Fig. 7). The use of the correct homokaryotic strain as control instead of the WT is, therefore, crucial for phenotypic analyses of *P. indica* transformants. In the presence of CR, the homokaryotic ^Prom*Pi*FGB1^FGB1:GFP strains had a considerably faster growth than the WT and the homokaryotic control strain (Fig. 3b). These results suggest that expression of FGB1 leads to altered cell wall properties in *P. indica* and *U. maydis* with an increased resistance to CR and further substantiate the involvement of this lectin in β-glucan biochemistry.

In order to test if expression of FGB1 alters cell wall composition, nuclear magnetic resonance (NMR) analyses of total acid hydrolysate of insoluble protein-free cell wall preparations of *U. maydis* and *P. indica* strains were performed. Cell walls of the FGB1:GFP *U. maydis* strain grown in YEPS-light (yeast extract, peptone, sucrose) had an altered glucose to *N*-acetylglucosamine ratio compared with the control GFP *U. maydis* strain (Supplementary Fig. 8). A second copy of FGB1 under the control of its own promoter in *P. indica* grown in CM where FGB1 production is induced, also led to an altered glucose to *N*-acetylglucosamine ratio compared with the homokaryotic *P. indica* control strain (Supplementary Fig. 8). This suggests that expression of FGB1 leads to changes in levels of β-glucans and/or chitin in the fungal cell wall (Supplementary Fig. 8). In line with an altered cell wall composition, ^Prom*Pi*GPD^FGB1:GFP *U. maydis* strains were significantly more sensitive to protoplastation by chitinases than the GFP controls (Fig. 3a). Thus, FGB1 affect cell wall composition and susceptibility to cell wall stress. This, however, does not explain why a large proportion of FGB1 is secreted into the culture filtrate (Supplementary Figs 1c and 3c).

### FGB1 suppresses β-glucan triggered ROS production in plants

**Figure 4:**
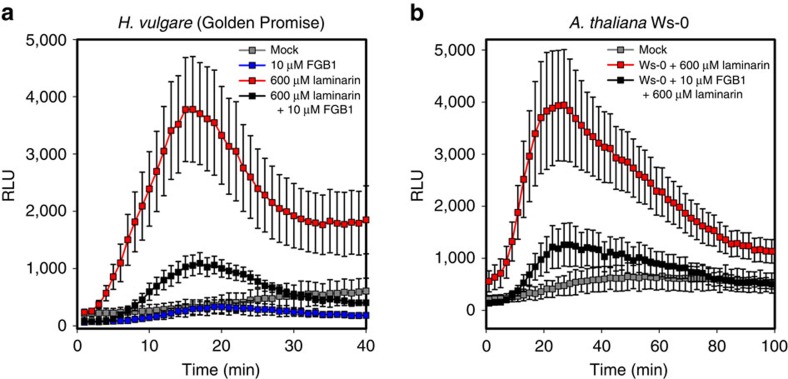
FGB1 supresses β-glucan-induced oxidative burst in barley and *A. thaliana* Ws-0. (**a**) Barley leaf disks react with a strong ROS burst after elicitation with 600 μM complex laminarin (red square). Incubation with 10 μM FGB1 supress ROS burst (black square). Neither 10 μM FGB1 (blue square) nor the mock water control (grey square) induce ROS production. (**b**) Leaf disks of *A. thaliana* ecotype Ws-0 react with ROS production after elicitation with 600 μM laminarin. Comparable to barley, Ws-0 ROS production was supressed by 10 μM FGB1. At least four independent repetitions with three different FGB1 batches were carried out and showed similar results. Error bars represent the standard error of the mean of 8–12 technical replicates (see Supplementary Fig. 10 for further details).

Two distinct chitin-binding lectins from *Cladosporium fulvum* (Ascomycota, Capnodiales), a pathogen of tomato, were shown to be involved in fungal cell wall protection against hydrolysis by plant chitinases in the case of Avr4, or to prevent chitin-triggered immunity *in planta* in the case of the abundantly secreted LysM-effector Ecp6 (refs 5, 7, 8). Plants secrete glucanases into the apoplast upon fungal attack, causing the release of glucan oligosaccharides that like chitin may act as MAMPs, such as the β-1,6-1,3-heptaglucan derived from the plant pathogen *Phytophthora sojae* (Stramenopiles, Oomycetes, Peronosporales)^19^,^20^. Therefore, we analysed the plant proteins present in the apoplastic fluid (APF) of barley roots obtained at 5, 10 and 14 days post inoculation (dpi) with *P. indica*. No barley endo-1,3-β-glucosidases were detected at 5 dpi. Among the most prevalent plant proteins present at 10 and 14 dpi we could detect two and four barley endo-1,3-β-glucosidases, respectively (Supplementary Table 1). This correlates with the expression pattern of FGB1 during root colonization (Fig. 1a). Recently, it was shown that β-glucan is required for cell wall rigidity in appressoria and fast-growing necrotrophic hyphae of *Colletotrichum graminicola* in maize but its synthesis is rigorously downregulated during biotrophic development possibly representing a strategy for evading β-glucan–triggered immunity^21^. To test whether FGB1 is able to protect β-glucan from glucanase activity *in vitro*, we incubated an endo- and an exo-β-1,3-D-glucanase, from barley (Sigma) and *Trichoderma virens* (Sigma) respectively, with laminarin in the presence and absence of native FGB1. No significant protection of the glucan polymer from glucanase activities was observed upon addition of FGB1 (Supplementary Fig. 9). We thus speculated that the large secreted portion of free FGB1 might be involved in mediating symbiosis through perturbation of glucan-triggered host immunity in different hosts. The ability of plants to respond to β-glucan elicitors is not universal and different plants recognize different glucan polymers^10^. To clarify whether β-glucan acts as an elicitor in barley and *Arabidopsis*, we used a luminol assay to measure the production of ROS upon incubation of plant tissues with laminarin. Barley root and leaf tissues and *Arabidopsis* leaves of the accession Ws-0, the latter previously identified as being insensitive to the bacterial MAMP flg22 because of a premature stop codon in the FLS2 kinase domain^22^, responded to laminarin with production of ROS (Fig. 4, Supplementary Fig. 10a,b,c,d,e). The laminarin-triggered ROS production is slightly delayed compared with that observed after chitin elicitation. This is most evident in barley (Supplementary Fig. 10e) and could be caused by the inability of the polymer laminarin to diffuse quickly in plant tissues or the need of hydrolysis by glucanases in the adequate elicitor before detection.

**Figure 5:**
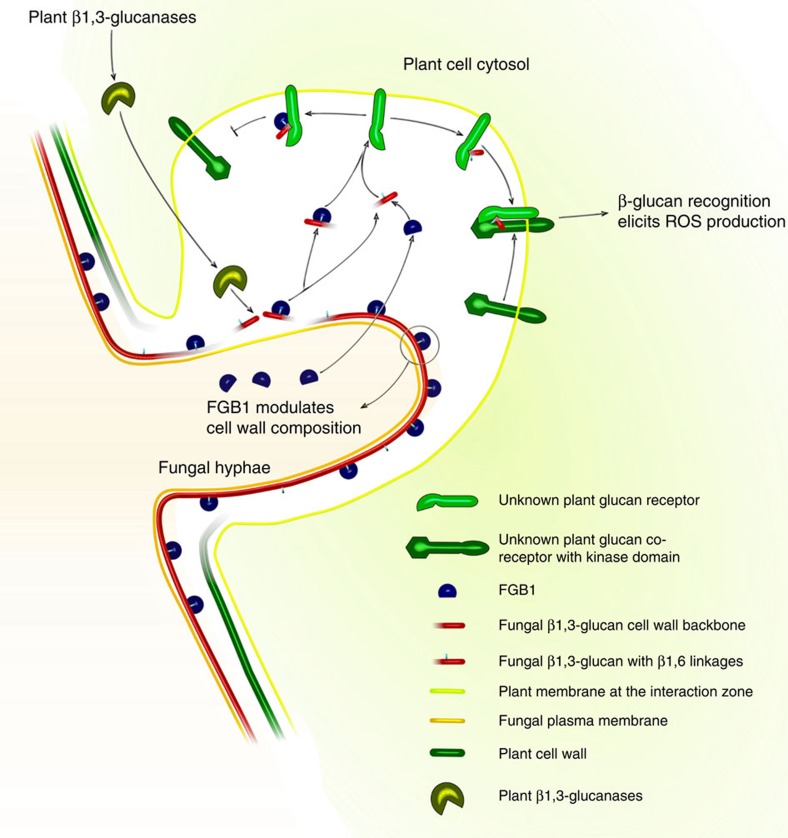
Model displaying the potential dual function of FGB1. Binding of FGB1 to the fungal cell wall (CW) after secretion is mediated through β-1,6-glycosidic linkages. FGB1 modulates CW polysaccharides composition. FGB1 does not protect the CW against β-1,3-endo- and β-1,3-exo-glucanase activities. Host glucanases activity release β-glucan fragments that can be sensed by a specific plant β-glucan receptor complex. Recognition leads to activation of basal defence mechanisms, such as the production of reactive oxygen species (ROS). Based on the observation that FGB1 is able to suppress β-glucan induced ROS production at substoichiometric concentrations, we hypothize that FGB1/β-glucan complexes have higher affinities to the plant β-glucan receptors than free β-glucan fragments. Binding of FGB1/β-glucan complexes to the β-glucan receptor would possibly prevent receptor/co-receptor association and defense downstream signalling.

To test whether FGB1 might alter glucan perception we measured laminarin-induced ROS production in the presence and absence of FGB1 in both barley and *Arabidopsis*. Treatment of plant tissue with laminarin resulted in strong ROS production, whereas addition of substoichiometric amounts of FGB1 significantly reduced this response (Fig. 4, Supplementary Fig. 10). The inhibition of ROS production occurred in a dose-dependent manner (Supplementary Fig. 10). In contrast, the host-translocated effector-protein Avr3a from *Phytophthora infestans*^23^, which like FGB1 is a SSP but does not act in the apoplast, and the recombinant (His)_6_FGB1^19–104^, which displays a different conformational change upon binding to laminarin (Supplementary Fig. 4d) did not affect the laminarin-induced ROS production (Supplementary Fig. 10a,g). The presence of *P. indica* in the roots of barley leads to suppression of the laminarin-triggered ROS production at 3, 5 and 9 dpi demonstrating the general ability of *P. indica* to suppress immunity (Supplementary Fig. 10f). To confirm the capacity of FGB1 to suppress β-glucan-mediated oxidative burst in different hosts, luminol-based ROS assays in *Nicotiana benthamiana* using flg22 or laminarin in presence and the absence of FGB1 were performed in an independent laboratory. FGB1 specifically suppressed the laminarin induced ROS production also in this plant system, but did not affect the response to flg22 (Supplementary Fig. 10h). The high efficiency of the laminarin-mediated oxidative burst suppression by FGB1, however, suggests that the perturbation by this novel glucan-binding lectin is possibly not established through glucan sequestration but conceivably through interference with component of the host immunity (Fig. 5), as proposed for the chitin-binding LysM-effector Ecp6 (ref. 8). The identification of the β-glucan receptor will aid in elucidating the molecular mechanisms behind the proposed function of FGB1 as suppressor of glucan-mediated immunity.

### FGB1 increases *P. indica* colonization rates in barley

In support of a role in colonization, overexpression of *Pi*FGB1 in *U. maydis* led to significantly increased virulence in maize (Supplementary Fig. 11). However, we observed no differences in colonization by ^Prom*Pi*FGB1^FGB1:GFP *P. indica* strains compared with the control strain (Fig. 3b). Perhaps this is due to the expression profile of FGB1:GFP which is similar to that of the endogenous FGB1 copy still present in the transformants. Also the GFP fusion at the C terminus of FGB1 may alter protein folding or function *in planta* decreasing its ability to interfere effectively with the host immune system. In order to test if the presence of the native FGB1 at an earlier time point of interaction would affect plant colonization, 9 μM of purified FGB1 was added to barley roots simultaneously to *P. indica* inoculation. This led to a fourfold higher colonization in all eight independent biological replicates (Fig. 3c).

## Discussion

To date, only few historical studies have focused on the role of fungal-derived glycan MAMPs other than chitin, highlighting the gap that exists and needs to be filled. Our data shows that the plant responsive lectin FGB1 from the root endophyte *P. indica* is abundantly secreted and possesses a so far uncharacterized protein domain conserved among fungi. We demonstrated that FGB1 is able to specifically interact with β-1,6-linked glucan, has the potential to alter cell wall composition and to confer resistance to the glucan-associated cell wall stressor CR. Furthermore, we showed that FGB1 is able to efficiently suppress laminarin-triggered ROS production in different hosts, thus representing a possible strategy to avoid β-glucan triggered immunity *in planta* and indicating a dual functionality of this protein. The striking functional similarities between chitin-binding LysM effectors and the glucan-binding FGB1 lectin indicate convergent mechanisms that safeguard different fungal cell wall-derived polymers from the plant immune system. Altogether these data provide fundamental evidence that β-glucan is an important fungal MAMP and that pathogenic, as well as mutualistic fungi need to protect β-glucan polymers from recognition *in planta*.

## Methods

### *P. indica* culturing conditions

*P. indica* (DSM11827, Deutsche Sammlung von Mikroorganismen und Zellkulturen, Braunschweig, Germany) was grown in complete medium (CM)^24^ or in YNB medium (1.7 g l^−1^ YNB without amino acids containing 20 g l^−1^ glucose). Liquid cultures were grown at 28 °C under constant shaking (130 r.p.m.). Propagation on solid medium was done at 28 °C on CM supplemented with 1.5% agar.

### Plant growth conditions and fungal inoculation procedures

*Arabidopsis* seeds were surface sterilized with 70% ethanol for 10 min, 100% ethanol for 7 min, germinated 3 days at 4 °C and subsequently grown for 7 days on half-strength Murashige and Skoog (½ MS) medium including vitamins (4.4 g MS salt per liter containing 4 g Gelrite, Duchefa) with a day : night cycle of 16 h : 8 h (light intensity, 47 μmol m^−2^ s^−1^) at 24 °C. All *Arabidopsis* experiments were performed with three biological replicates. For each biological replicate 3 plates containing 20 plants were pooled. For colonization studies ca 0.3 ml of chlamydospore suspensions (500,000 spores ml^−1^ in 0.002% Tween20 aqueous solution) were applied to the roots of each plant of 7-day old germlings.

Barley seeds (*H. vulgare* L. cv Golden Promise) were surface sterilized with 70% ethanol for 1 min followed by 1.5 h 12% sodium hypochlorite sterilization before being washed with sterile distilled water (3 h). Sterilized seeds were kept sterile and in the dark for 3 days on wet filter paper at room temperature. Subsequently, germlings were grown in sterile jars containing (1/10 PNM (plant nutrition medium; ref. 11) and cultivated in a growth chamber with a day : night cycle of 16 h : 8 h (light intensity, 108 μmol m^−2^ s^−1^) and temperature of 22 °C 18 °C. For colonization studies 1 ml of chlamydospore suspensions (500,000 spores ml^−1^ in 0.002% Tween20 aqueous solution) was applied to the roots of 3-day-old barley germlings. Tween water-treated germlings were used as control. For colonization experiments in presence of purified FGB1, a sterile filtrated protein stock solution (in water) was added to the spore solution and to an additional mock control at a final concentration of 9 μM. Root samples were collected at the indicated times after inoculation and carefully washed in distilled water. Roots were cut and the first 4 cm were harvested and immediately frozen in liquid nitrogen. All barley experiments were prepared with three to eight biological replicates with four plants per jar, and two to three independent replicate experiments.

### Preparation of *P. indica* secreted protein fractions

To analyse *P. indica* secreted proteins the culture filtrate of 1 week old cultures were first filtrated through cell strainers with a 40 μm cutoff before centrifugation for 15 min at 10,000*g*. Five millilitre 95% trichloroacetic acid was added to 20 ml cell free culture filtrate and incubated overnight at 4 °C. Subsequently, 25 ml of 100% acetone was added and the solution was centrifuged for 30 min at 10,000*g*. Protein pellets were washed with 100% acetone and dried before boiling in Laemmli SDS sample buffer containing 8 M urea and 2 M thiourea.

### Enrichment of secreted proteins with lectin like properties

Two liters of culture filtrate of 7-day old *P. indica* cultures were harvested by filtration through Miracloth (Merckmillipore) and subsequent filtration through 0.22 μm sterile nitrocellulose filters. The obtained cell free culture filtrate was pH adjusted with phosphate buffer to pH 7.5 and separated via 40 ml Fractogel-EMD-SO^3-^ (M) (Merck) and EMD TMAE Hicap (M) (Merck) ion exchange material into acidic- and alkaline-protein fractions. The obtained protein fractions were dialysed against 10 mM sodium acetate buffer containing 150 mM NaCl pH 5.0 and 400 μl were incubated with 50 μl sepharose beads for 30 min at room temperature before washing with an excess of binding buffer. Subsequently, the sepharose beads were boiled in Laemmli SDS-buffer and bound proteins were analysed by SDS–polyacrylamide gel electrophoresis (PAGE).

### Purification of native FGB1 PIIN_03211

To purify native FGB1 the supernatant of 7-day old *P. indica* cultures (2 l) grown in CM was filtrated through Miracloth (Merckmillipore) and subsequently filtrated through 0.22 μm sterile nitrocellulose filters. The obtained cell free culture filtrate was diluted 1:1 with water and supplemented with 1 mM phenylmethylsulphonyl fluoride and 15 ml 1 M sodium acetate buffer pH 5.0 per 4 l to raise the pH of the culture to pH ∼5. The culture filtrate was then applied to columns containing EMD TMAE Hicap (M) (Merck) ion exchange material. The flow through of this step was then applied to columns containing Fractogel-EMD-SO^3-^ (M) (Merck) ion exchange material. After protein binding the column was washed with 10 volumes of 10 mM sodium acetate buffer pH 5.0. The crude FGB1 concentrate was obtained by step elution of bound proteins with 10 mM sodium acetate buffer pH 5.0 containing 1.5 M NaCl. Final purification was done by applying the SO_3_^-^ eluate to a spin concentrator with a 30,000 Da cutoff. The flow through of this step was then concentrated using a 5,000 Da cutoff spin concentrator and the concentrated protein was 2 × dialysed (1 × 3hr, 1 × overnight) against 3 l of water.

### Analysis of FGB1 binding to cell wall preparations

To assess the binding capability of FGB1 to protein-free cell wall preparations of barley roots (17-days old), *F. oxysporum* (6-day old mycelium) and *P. indica* (6-day old mycelium), the plant and fungal samples were grinded in liquid nitrogen. Subsequently, water was added to the powder and the suspensions were centrifuged for 15 min at 8,000*g*. The resulting pellets were homogenized using an UltraTurax with 8 M urea containing 2% SDS and incubated for 2 h at 80 °C. This was followed by centrifugation for 15 min at 8,000*g*. The supernatant was removed and the pellets were homogenized with 10 mM sodium phosphate buffer pH 7.5 and centrifuged again. This step was repeated 3–4 times. Subsequently, the pellets were homogenized with PBS containing 0.3 mg ml^−1^ Proteinase K and incubated overnight at 37 °C. The cell wall polysaccharides were pelleted, homogenized in 2% SDS+8 M Urea and incubated at 80 °C for 2 h before centrifugation. The supernatant was removed and the pellets were homogenized in water and centrifuged again (3–4 repeats). The pellets were then homogenized 2 × with 100% acetone and pelleted before drying at 80 °C. Prior use the obtained polysaccharide pellets were grinded to a fine powder.

For polysaccharide pull downs 10 mg powder was weighted into test tubes and 500 μl water was added. The polysaccharides were then sonicated for 30 s before the addition of 200 μl protein solution to obtain a final buffer conditions of 10 mM sodium acetate pH 5.0 containing 300 mM NaCl and 20 μM FGB1. The mixture was incubated for 1 h at room temperature before centrifugation for 10 min at 17,000*g*. Supernatant fractions were obtained by removal of 600 μl clear protein solution that was precipitated with 500 μl 40% trichloroacetic acid overnight at 4 °C. The supernatant samples were centrifuges for 30 min at 17,000*g* (4 °C) and the pellets were washed 2 × with 100% acetone before drying. SDS–PAGE samples were obtained by boiling of for 10 min at 100 °C with 100 μl Laemmli SDS sample buffer containing 8 M urea and 2 M thiourea and 7.5 μl were loaded onto SDS gels. Pellet fractions were washed 3 × with buffer. One-hundred microlitres of protein sample buffer was added to the wet pellets and boiled. Fifteen microlitres of the pellet fractions were loaded onto SDS-gels for analysis.

### Database searches and sequence alignments

BlastP search with the FGB1 amino acid sequence was performed against Genbank nr database (1.0E-3) and JGI MycoCosm (1.0E-3). Alignment was performed with Muscle using Mega6 (ref. 25) and visualized using Maestro 10.3 (Schroedinger Inc.).

### DNA and RNA extraction and cloning procedures

DNA was extracted from ∼200 mg of ground material. The powder was incubated for 5 min at room temperature under slight rotation with 500 μl CTAB buffer (2% hexadecyl trimethyl-ammonium bromide, 100 mM Tris/HCl, 20 mM EDTA, 1.4 M NaCl and 1% polyvinyl pyrrolidone vinylpyrrolidine homopolymer Mw 40,000, pH 5.0). Subsequently, 250 μl chloroform: isoamyl-alcohol (24:1) was added followed by an additional incubation for 5 min at room temperature and centrifugation. The water phase was collected and polysaccharides were precipitated using ethanol before final DNA precipitation using 1 volume of isopropanol. Total RNA from 200 mg of ground material was extracted with TRIzol (Invitrogen, Karlsruhe, Germany) following the manufacturer's instructions followed by complementary DNA (cDNA) synthesis (First Strand cDNA Synthesis Kit, Fermentas). Real-time quantitative PCR analyses were performed with 10 ng of DNA or cDNA mixed with the appropriate primers in 15 μl of SYBR green Supermix (Bio-Rad) using the following amplification protocol: initial denaturation for 10 min at 95 °C, followed by 40 cycles of 30 s at 95 °C, 30 s at 59 °C, 30 s at 72 °C and a melt curve analysis. The relative DNA amount or relative expression and its fold change values were calculated using the inline image method. The FGB1 sequence containing the signal peptide and lacking the stop codon was amplified from cDNA and cloned into pGoGFP using the ClaI and HindIII restriction sites. The FGB1 gene was in frame with a C-terminal GFP sequence in order to produce the FGB1:GFP fusion protein when transcribed and translated. In order to substitute the GPD-promoter sequence of pGoGFP with that of FGB1 the GPD promoter was removed using the ApaI and ClaI restriction sites. Part of the FGB1 promoter was amplified using the primers 5′-AACGTGCGGCCGCTGAAT-3′ and 5′-CGATAGAGTTGGAGGCAAATC-3′ and fused to the remaining nucleotide sequence obtained from GenScript. The whole construct was then cloned into pGoGFP using the ApaI and ClaI restriction sites. All used primer sequences are listed in Supplementary Table 2.

### FGB1 expression analyses

To account for the different spore germination rates in CM and YNB medium mycelium of one week old *P. indica* WT culture grown in CM (50 ml) was filtrated and washed with 0.9% NaCl before crushing in 50 ml liquid CM. Subsequently, the mycelium was regenerated for 2 days. After regeneration the mycelium was filtrated and washed again and 0.5 g was used to inoculate either 25 ml CM or YNB medium (three replicates each). Cultures were grown for the indicated times at 28 °C before mycelium was harvested and RNA/cDNA preparation and quantitative PCR were performed.

### Confocal microscopy

Images were recorded using a Leica TCS SP8 confocal microscope. GFP imaging was done with an excitation at 488 nm and detection of the emitted light between 500–540 nm. FM4-64 (Molecular Probes, Karlsruhe, Germany) staining was carried out for 5 min after addition of 1 μl of a 2 mM stock solution dissolved in dimethylsulphoxide per 1 ml of culture and imaging was done using an excitation wavelength of 561 nm and detection between 580–660 nm. For wheat germ agglutinin-Alexa Fluor 594 (WGA-AF594, Invitrogen) 2 μl of a 1 mg ml^−1^ stock solution (dissolved in PBS) was used in a volume of 0.5 ml (excitation at 561 nm detection at 610–650 nm).

### *U. maydis* plasmolysis and cell wall stress assay

The solopathogenic haploid *U. maydis* strain SG200 (a1 mfa2 bE1bW2) was used in this study for transformation and localization studies of *Pi*FGB1:GFP. In general, the SG200 transformant strains were grown in liquid YEPS-light (0.4% yeast extract, 0.4% peptone, 2% sucrose) containing 5 μg ml^−1^ hygromycin at 28 °C. For plasmolysis and drop dilution tests 36 h old cultures grown in YEPS-light supplemented with 5 μg ml^−1^ hygromycin were diluted to OD_600_∼0.4 and grown to OD_600_ ∼0.8. For plasmolysis, the corresponding cultures were diluted 1:1 with 3 M NaCl and immediately imaged. For drop dilution tests, 1 ml of the respective cultures were pelleted and adjusted with water to OD_600_=1.0 before preparing five successive 1:10 dilutions. Six microlitres of each dilution was dropped onto the respective plates. Plates were incubated for the indicated times at 28 °C. Charcoal plates (potato dextrose agar Sigma containing 1% charcoal) were used to assess filamentation and kept at room temperature for the indicated times.

### *U. maydis* protoplastation

Overnight cultures of SG200 expressing either FGB1:GFP or GFP inoculated with identical inoculum were adjusted to an OD_600_ of 1 and 1 ml was pelleted. The pellets were resuspended in 50 μl 20 mg ml^−1^ chitinase (*Trichoderma viride*; Sigma C8241-25UN) dissolved in 20 mM sodium citrate, 1 M sorbitol, pH 5.8. The samples were incubated for 15 min at 37 °C and the reaction was stopped by placing the samples on ice and images were taken immediately. Protoplasted and non-protoplasted cells were counted in three technical replicates for each clone.

### Maize infection assays for *U. maydis*

Seven-day old maize seedlings were infected with the respective *U. maydis* SG200 transformant strains^26^ either expressing FGB1:GFP or GFP under the control of the *P. indica* GPD promotor (plasmid pGoGFP). Disease symptoms were scored 12 dpi according to Djamei *et al*.^26^ for three independent isolates each and three biological replicates.

### Isothermal titration calorimetry

Isothermal titration experiments were performed to analyse the affinity of FGB1 to different soluble oligo- and poly-saccharides using a VP-ITC instrument at 20 °C. The instrument was heat-pulse-calibrated and the protein samples were extensively dialysed against water. Titrant stock solutions were prepared with the same batch of water as used for dialysis. All solutions used were degassed before filling the sample cell and syringe. Compound concentrations are given in the figure legends for the individual experiments. The ITC stirring speed was set to 300 r.p.m.; the feedback gain mode was set to medium. Since the initial injection generally delivers inaccurate data, the first step was omitted from the analysis. The collected data were analysed using the program ‘Origin' (MicroCal) and binding isotherms were fitted using the binding models provided by the supplier. Errors correspond to the s.d. of the nonlinear least-squares fit of the data points of the titration curve.

### Circular dichroism spectroscopy

In order to gain information about the FGB1 secondary structure in the presence and absence of different oligo- and poly-saccharides circular dichroism-spectra were recorded on a Jasco J700 in degassed water at 20 °C. Protein and ligand concentrations are given in the respective figure legends. All spectra were subtracted with the data obtained for the water blank.

### Oxidative burst assay

To test the effect of FGB1 on the laminarin-triggered H_2_O_2_ production, barley plants were grown for 6–8 days in soil in a phytochamber at 21 °C with 16 h light/8 h dark, 60% humidity and 190 μmol m^−2^s. *A. thaliana* Ws-0 plants were grown in soil under green-house conditions for three to four weeks. Leave discs were cut from barley and *Arabidopsis* plants with a biopsy puncher (diameter 5 mm), transferred to a 96-well plate, flooded with water and incubated for 16 h in the dark. To measure the oxidative burst in barley roots the seeds were surface sterilized and germinated for 3 days on wet filter paper in the dark. Subsequently, the germinated seeds were transferred in jars with 100 ml 1/10 PNM medium and grown for 4 days in a phytochamber at 21 °C with 16 h light/8 h dark, 60% humidity and 190 μmol m^−2^s. The first 4 cm of the main root was harvested, washed in water to remove residual medium and cut into 5 mm pieces. Three root pieces were added per well, which were flooded with water and incubated for 16 h in the dark. Before the assay the water was in all cases replaced by 10 mM MOPS buffer pH 7.4 containing 10 μM L-012 (Wako Chemicals) and 10 μg ml^−1^ horseradish peroxidase (Sigma Aldrich). The luminescence was measured for 100 min after the proteins were added and either laminarin (Sigma Aldrich), chitin, flg22 (Genscript) or water were injected. The measurement was conducted using a Tecan Infinite M200 Pro plate reader. ROS measurements with leaf disks of *N. benthamiana* (7 weeks old plants) were carried out in water in presence of 10 μM L-012 and 10 μg ml^−1^ horseradish peroxidase using a Photek photon counter.

### SDS–PAGE and western blotting

SDS–PAGEs were performed according to the manufacturer's instructions (Invitrogen). Here in brief, gradient 4-12% Bis-Tris NuPage gels were used with NuPage MES-SDS running buffer (Invitrogen). Protein samples were dissolved in Laemmli SDS buffer containing 8 M urea, 2 M thiourea and 2% β-mercaptoethanol. For western blotting protein from unstained gels were transferred onto nitrocellulose membranes using a semi dry blotting system from Biometra. Anti GFP antibody was obtained from Roche (#11814460001) and used at a 1:2,000 dilution. As secondary antibody an anti-mouse antibody purchased from Sigma (A2304) was used at a dilution of 1:2,000.

### Thin-layer chromatography and glucanase activity assay

In order to test whether FGB1 is able to protect β-glucan from hydrolysis by glucanases an endo-β1,3-D-glucanase and an exo-β1,3-D-glucanase, from barley and *T. virens*, respectively, were incubated with laminarin in the presence and absence of FGB1. After mixing of the individual compounds (concentrations are given in the figure legend) the reactions were kept at 37 °C (700 r.p.m. shaking) for 15 min in case of the reaction assays containing the endo-glucanase and 1 h in case of the exo-glucanase. In all, 4 × 1.5 μl assay were spotted per sample on silica-gel 60 F_254_ plates (Merck, 1.16834). TLC was performed in *n*-butanol-isopropanol-water (3:12:4). After the run the plates were dried at room temperature and developed by spraying with 1-napthol (15 mg of napthol in 12.4 ml ethanol+1 ml H_2_O+1.6 ml H_2_SO_4_) and subsequent incubation at 200 °C (ca. 30 s). Barley endo-β1,3-D-glucanase (E-LAM HV) and *T. virens* exo-β1,3-D-glucanase (E-EXBGTV) were obtained from Megazyme.

### Bicinchoninic acid assay

Laminarin (from 0.6 to 5 g l^−1^=8.53 μM to 1 mM) was incubated with either exo-1,3-β-D-glucanase (*T. virens*, Megazyme E-EXBGTV) in sodium acetate buffer (100 mM, pH 4.5) or endo-1,3-β-D-glucanase (barley, Megazyme E-LAMHV) in sodium acetate buffer (100 mM, pH 5.2) (12,5 or 25 U l^−1^) at 40 °C and 900 r.p.m. (Eppendorf Thermomixer) and FGB1 (5.83 to 42.53 μM) was added. Samples were taken at different time intervals and the reaction was stopped by heating the samples at 95 °C for 5 min. The released glucose was quantified with the bicinchoninic acid (BCA)-assay via the optical density at 540 nm with a 96-well plate reader (TECAN Sunrise) using glucose as a standard. The BCA assay was performed according to McFeeters *et al*.^27^. In brief, this assay relies on the proportional reduction of Cu^2+^ (from CuSO_4_) to Cu^+^ (biuret reaction) by the reducing sugars in solution. Colorimetric quantification was performed subsequently to the chelation Cu^+^ by BCA sodium salt (1:2 stoichiometry). The formed complex strongly absorbs light at a wavelength of 540–560 nm in an alkaline environment.

### Determination of the glucose to *N*-acetyl glucosamine ratio

Deuterated hydrochloric acid (37% DCl in D_2_O) was used to catalyse the acidic hydrolysis of polymers from the non-soluble fraction of the cell wall to their monomers for 120 min at room temperature^28^. The ratio of glucose and *N*-acetyl glucosamine was determined after hydrolysis by the distribution via ^1^H-NMR-spectroscopy (Bruker DMX 600 FT-NMR-spectrometer; ^1^H: 600 MHz at 295 K). The NMR-spectra were calibrated by setting the water signal to 9.10 p.p.m. The amount of *N*-acetylglucosamine was calculated using the integrated signal of the acetylic group at 2.26 p.p.m. The amount of glucose was calculated from the sum of all anomeric protons minus that of the *N*-acetylglucosamine. The ^1^H-NMR exhibited three anomeric protons at 5.03 p.p.m., at 4.50 p.p.m. (α-D-glucose, β-D-glucose, *N*-acetylglucosamine) and a third signal at 5.98 p.p.m. The different samples had a dry mass weight between 10 to 25 mg and were dissolved in 750 μl DCl. Only clear solutions were measured by NMR. Each measurement took about 5 min. To assess the de-*N*-acetylation rate of *N*-acetylglucosamine we performed control experiments with chitin (Roth, Art. Nr. 8845.1) glucose and *N*-acetylglucosamine, all dissolved in concentrated DCl (37%, 9.8 M). The control reactions were followed for up to 22 h. We observed in accordance with Einbu and Varum^28^ a maximal de-*N*-acetylation of 2% in the samples after 22 h. After 2 h the hydrolysis rate was <0.5%.

In detail: The ^1^H-NMR spectrum of chitin showed anomeric-protons between 5.1 and 4.3 p.p.m. with an integral of 1H. The protons of the acetyl group resonated at 2.3 p.p.m. with an integral of 3H. Thus, the ratio of the anomeric protons and the acetyl-group signals were 1:3 which is in agreement with chitin consisting of *N*-acetylglucosamine units. Control NMR measurements were also performed with chitin after 30 min, 2 and 22 h in DCl. There, the ratio of the anomeric protons and acetyl protons was still 1:3. To assess the effect of non-complete polysaccharide hydrolysis the signals of the acetyl protons of *N*-acetylglucosamine from oligomers, dimers, the oxazolium ion were measured. All resonated at around 2.2 p.p.m. and no significant hydrolysis of the acetyl group was observed within 2 h under the experimental conditions for the tested saccharides. After 22 h, 2% of the acetyl groups of *N*-acetylglucosamine were found to be hydrolysed. Because beta-glucan does not have acetyl groups and chitin and their degradation products can be distinctly measured by NMR after 120 min and did not show any significant de-*N*-acetylation, the ratio between beta-glucan and chitin can be analysed. Glucose was also measured as a control over 23 h. Here the integrals of the anomeric protons (6.41–4.45 p.p.m.) to the remaining signals (3.7–3.0 p.p.m.) were 1:6.3. Considering the spectra of glucose, *N*-acetylglucosamine and Chitin from 0–22 h we concluded that the observed signals were stable and appropriate to determine the ratio of chitin and beta-glucan.

### APF collection from roots colonized by *P. indica*

Barley seeds (*H. vulgare* L. cv Golden Promise) were surface sterilized for 1 min under light shaking in 70% ethanol followed by 1 h in 12% sodium hypochlorite. Subsequently, seeds were washed repeatedly for 1 h with sterile distilled water. After germination for 3 days at 22 °C in complete darkness on wet filter paper, germinated seeds were transferred into jars with 1/10 PNM under sterile conditions and inoculated with 3 ml of chlamydospores (concentration 500,000 spores ml^−1^) of *P. indica* GoGFP strain^24^ and cultivated in a growth chamber with a day: night cycle of 16 h: 8 h (light intensity, 108 μmol m^2^ s^−1^) and temperature of 22 °C 18 °C. For APF extraction three time points reflecting different interaction stages have been chosen: Early and late biotrophic phase (5 and 10 dpi), as well as cell death associated phase (14 dpi). After respective growth time, roots were thoroughly washed and cut into pieces of 2 cm length (second to fourth centimetre of the root). Samples were vacuum infiltrated (Vacuumbrand, CVC 3000, VWR) with deionized water 5 × 15 min at 250 mbar with 1 min atmospheric pressure break. Bundled infiltrated roots were centrifuged in 5 ml syringe barrels at 2,000 r.p.m. for 15 min at 4 °C. Pooled APF was stored at −20 °C. In order to exclude, cytoplasmic contamination APF was tested in immunoblots with GFP antibody. For higher peptide mass accuracy APF was deglycosylated with Protein Deglycosylation Mix (P6039S, New England BioLabs, Ipswich, USA) under denaturing conditions. For peptide mass, identification samples were digested with trypsin and subjected to liquid chromatography–MS/MS mass spectrometry. Proteins matched to peptides were analysed for the presence of signal peptides and exclusiveness in the three specific time points of APF extraction.

### Data availability

Data supporting the findings of this study are available within the article and its Supplementary Information files and from the corresponding author on reasonable request.

## Additional information

**How to cite this article:** Wawra, S. *et al*. The fungal-specific β-glucan-binding lectin FGB1 alters cell-wall composition and suppresses glucan-triggered immunity in plants. *Nat. Commun.* **7,** 13188 doi: 10.1038/ncomms13188 (2016).

## Supplementary Material

Supplementary Information Supplementary Figures 1-11 and Supplementary Tables 1 and 2
