# Supplementary material for: The fungal-specific β-glucan-binding lectin FGB1 alters cell-wall composition and suppresses glucan-triggered immunity in plants
Source: Nat Commun. 2016 Oct 27;7:13188. doi: 10.1038/ncomms13188 (PMC5095285; doi:10.1038/ncomms13188)

Supplemental Material

Supplementary Figures

Supplementary Figure 1:

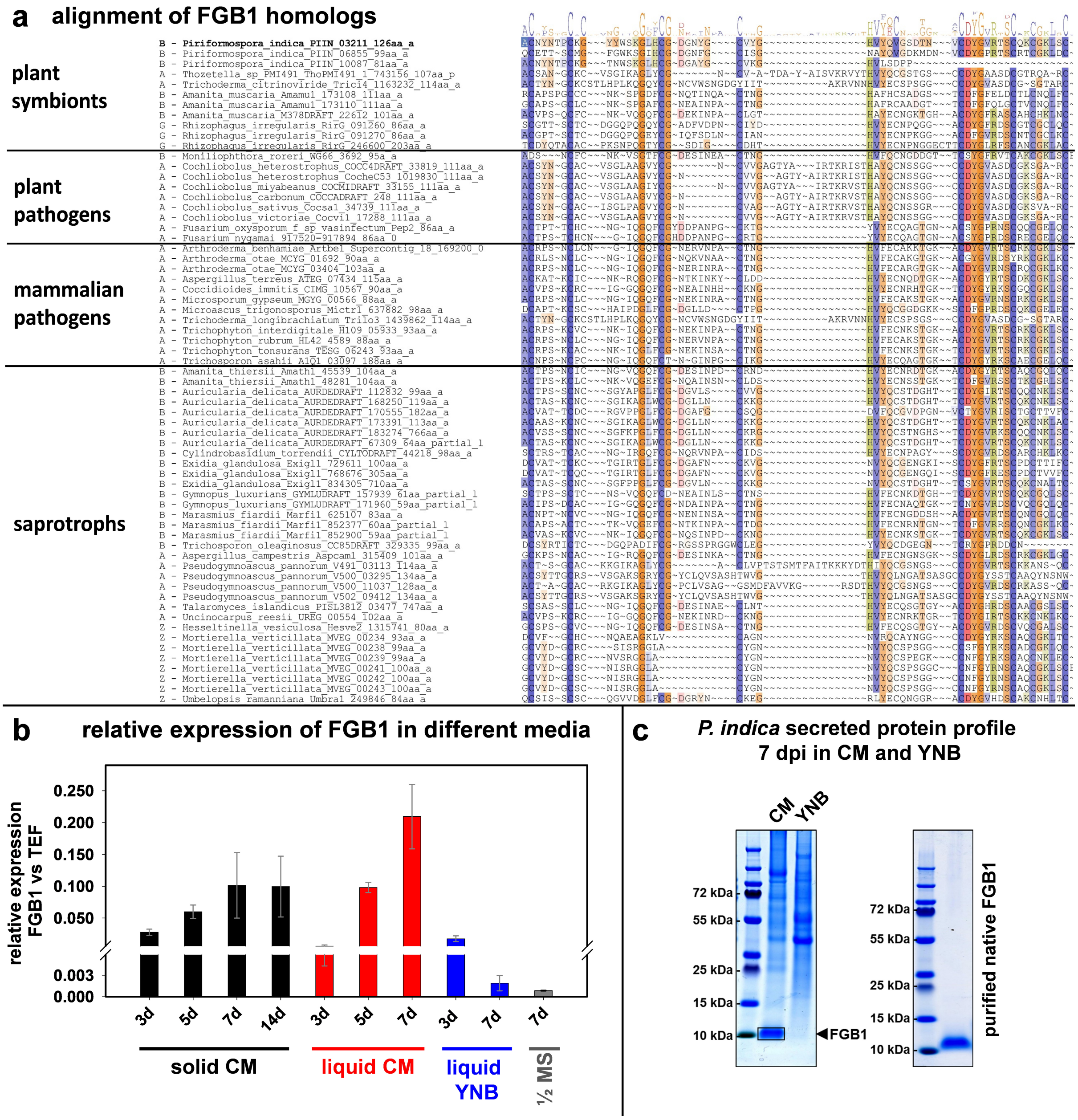

Supplementary Figure 1: FGB1 is a small secreted protein from *P. indica* that contains a novel lectin domain and is highly expressed during axenic growth in complex medium (CM) but not in yeast nitrogen base- (YNB) or ½MS-medium. **a** Protein sequence logo and alignment of FGB1 (PIIN_3211) to homologues from different fungi. BlastP search was performed against Genbank nr database (1.0E-3) and JGI MycoCosm (1.0E-3). Alignment was performed with Muscle using Mega6 (Tamura et al., 2013) and visualized using Maestro. B = Basidiomycota, A = Ascomycota, G = Glomeromycota, Z = Zygomycota. **b** *P. indica* FGB1 transcript levels in solid CM and solid ½MS medium as well as liquid CM and YNB medium. FGB1 transcript levels in liquid CM and YNB medium were obtained from fungal samples after inoculation of the respective media with a 7 day old *P. indica* WT culture mycelium grown in CM that was crushed and subsequently regenerated for 2 days in liquid CM. Samples were taken at the indicated times. The error bars are standard deviations of three biological replicate samples (8 technical replicates each). **c** CBB stained SDS-PAGEs showing the secreted

protein fraction of *P. indica* grown in liquid CM and YNB for 7 days. Right gel in c depicts a preparation of purified native FGB1. The rectangle indicates the protein band that was excised from the gel and subsequently identified by LC-MS/MS after tryptic digest as FGB1.

Supplementary Figure 2:

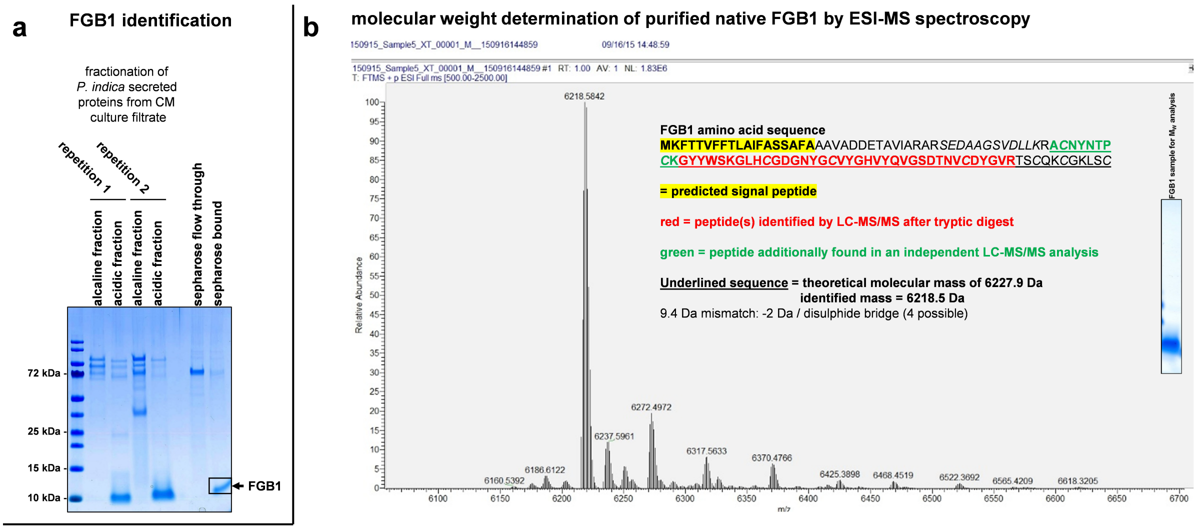

complete list of peptides identified after tryptic digest and subsequent LC-MS/MS analysis for the purified FGB1 sample used for molecular mass determination.

Accession	Description	Score	Coverage	# Proteins	# Unique Peptides	# Peptides	# PSMs	# AAs	MW [kDa]	calc. pI					
PIIN_03211_hypoth	PIIN_03211_hypothetical_orf	46.03	29.37	1	2	2	4	126	13.5	6.49					
	A3	Sequence	# PSMs	# Proteins	# Protein Groups	Protein Group Accessions	Modifications	ΔCn	IonScore	Exp Value	Charge	MH+ [Da]	ΔM [ppm]	RT [min]	# Missed Cleavages
	High	GYYWSK	3	1	1	PIIN_03211_hypothetical_orf		0.0000	24	0.003874265	2	803.37328	1.25	37.08	0
	High	GLHcGDGNYGcVYGHVYQVGSDT	1	1	1	PIIN_03211_hypothetical_orf	C4(Carbamidomethyl); C11(Ca	0.0000	20	0.010989299	3	3478.47141	2.28	68.77	0

Supplementary Figure 2: Native FGB1 is a small disulfide bridge-containing secreted protein with lectin like properties. **a** SDS-PAGE showing culture filtrate fractionation of *P. indica* secreted proteins after growth in CM into alkaline- and acidic-fraction (10 mM sodium phosphate pH 7.5). Subsequent binding to sepharose beads revealed one prominent small protein band bound to this matrix (indicated by a rectangle) that was excised and identified by LC-MS/MS as FGB1. **b** Molecular weight determination of purified FGB1 revealed a mature secreted protein with a molecular mass of 6,218.5 Da. LC-MS/MS analysis of the same sample identified peptides belonging to FGB1 with no additional peptides present in the preparation.

Supplementary Figure 3:

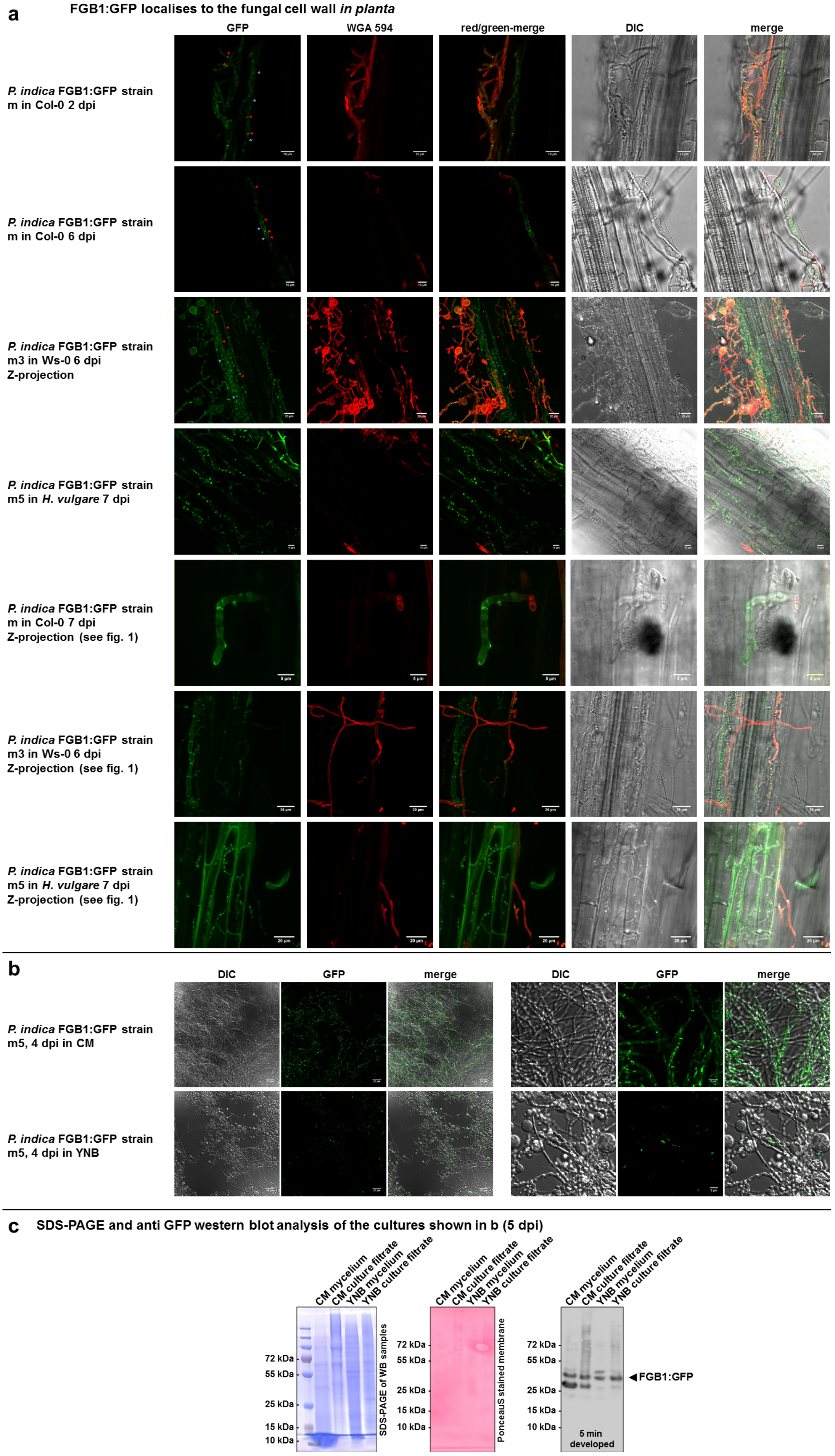

Supplementary Figure 3: FGB1:GFP fusion protein produced under the control of the native promoter localizes to the *P. indica* cell wall *in planta* and during axenic growth in liquid CM. **a** top panels: ^{PromFGB1}FGB1:GFP clone 7 localizes to the fungal septa (red arrowheads) and cell wall in colonized *A. thaliana* Col-0 and Ws-0 roots as well as barley roots. Intracellular hyphae cannot be stained with the chitin-binding WGA-AF594 dye (blue arrowheads). Chitin staining of intracellular hyphae has been shown with samples cooked in KOH prior WGA staining indicating that chitin is present in intracellular hyphae (*4*). The last three panels show the fluorescence- and DIC-channel of the images shown in figure 1, main text. **b** ^{PromFGB1}FGB1:GFP (strain m5) is expressed during axenic growth in CM but production is strongly reduced in YNB medium after 4 days of growth. FGB1:GFP expression was analyzed in liquid CM and YNB medium at the indicated times. Starting cultures were inoculated with crushed mycelium from *P. indica* FGB1:GFP strains from a 7 day old culture. **c** left: CBB stained SDS-PAGE of mycelium and culture filtrate samples used for anti-GFP western blot of the cultures analyzed by confocal microscopy shown in **b**. middle: Ponceau S stained nitrocellulose membrane. right: ECL developed anti-GFP western blot.

Supplementary Figure 4:

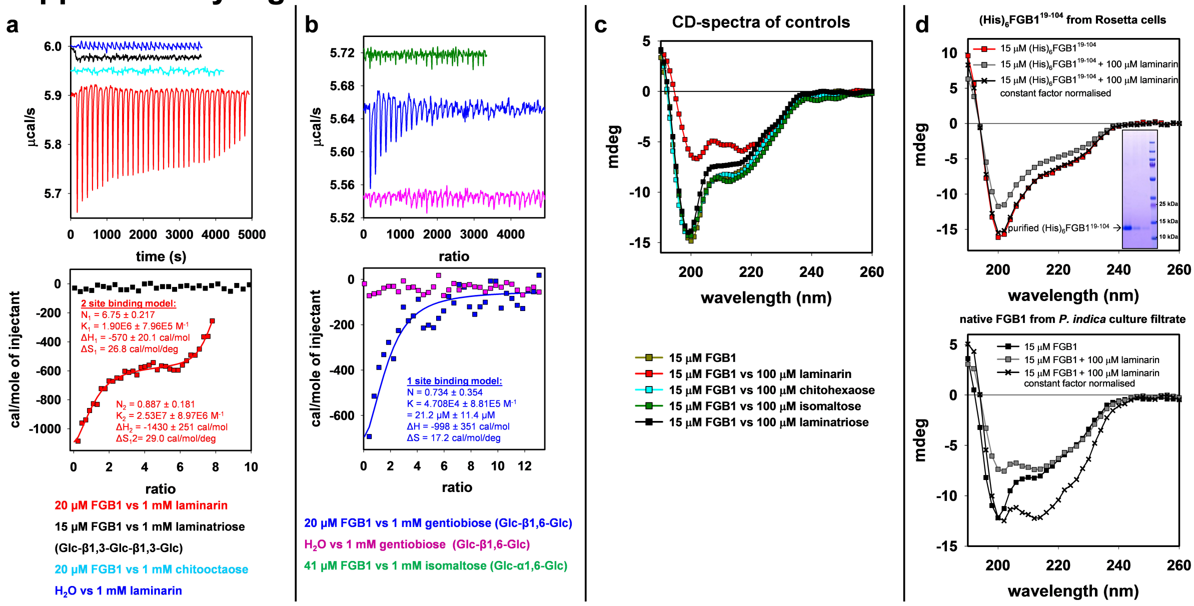

Supplementary Figure 4: FGB1 specifically binds to laminarin and gentiobiose. **a/b** raw data (upper pannels) and integrated heat values (lower pannels) for the titration of laminarin (in a, –■–) and gentiobiose (Glc-β1,6-Glc, in b, –■–) to FGB1. Experiments were performed at least 4 times with independent FGB1 preparations and resulted in similar thermodynamic values (see also figure 2, main text). Control titrations are shown for: laminarin vs water (in a, —), laminatriose vs FGB1 (in a, ––), chitooctaose vs FGB1 (in a, —), isomaltose vs FGB1 (in b, —) and gentiobiose vs water (in b, —). A first binding transition to laminarin appeared at a protein:ligand ratio of 1:1 with a corresponding binding constant of about 80 nM . A second transition occurred at a molecular ratio of 1:7 yielding a Kd value of about 800 nM. The observed second binding transition could be due to a molecule of FGB1 binding to a laminarin polymer where a molecule of FGB1 is already present. This can occur only in longer laminarin polymers where spacing and position of the β1,6-glycosidic bonds allows this second binding event to happen. The lower affinity could originate from reduced degree of freedom in the ligand caused by the already present FGB1 molecule and/or sterical hindrances between the two FGB1 molecules. Alternatively, the FGB1 glucan binding pocket could permit the binding of a second laminarin molecule with a lower affinity but taking the size of the molecule into account this is less likely. **c** CD-spectra recorded for 15 µM FGB1 (–■–) alone or in presence of 100 µM laminarin (–■–), 100 µM chitohexaose (–■–), 100 µM isomaltose (Glc-α1,6-Glc –■–) and 100 µM laminatriose (Glc-β1,3-Glc-β1,3-Glc –■–) with an independent FGB1 preparation to the results shown in figure 2 (main text). No significant shift in the secondary structure of FGB1 was observed in presence of chitohexaose, isomaltose or laminatriose. Spectra were substracted by the spectra obtained for the respective saccharides in water. **d** Top pannel: The whole CD-spectrum of 15 µM recombinant $(His)_6FGB1^{19-104}$ (–■–) shifts to lower millidegree values in presence of 100 µM laminarin (–■–). This shift is characterised by a constant factor and the whole spectrum can be overlayed to the spectrum in absence of laminarin by a respective multiplication (–x–). In addition, protein aggregates were observed after addition of laminarin to $(His)_6FGB1^{19-104}$ indicating that in *E. coli* produced FGB1 which lacks the disulfide bridges present in the native protein aggregates upon the structural changes induced by the binding of laminarin. Bottom pannel: In contrast, the CD-spectrum of 15 µM native FGB1 (–■–) only shows local spectral changes in presence of 100 µM laminarin (–■–). Normalisation factor was calculated by deviding the mdeg value at 200 nm of the protein spectra by the 200 nm value of the spectra obtained in the presence of laminarin. Before use the protein stock solutions were dialysed 2x against 3 l of water and the dialysis water was used to prepare the laminarin stock solution.

Supplementary Figure 5:

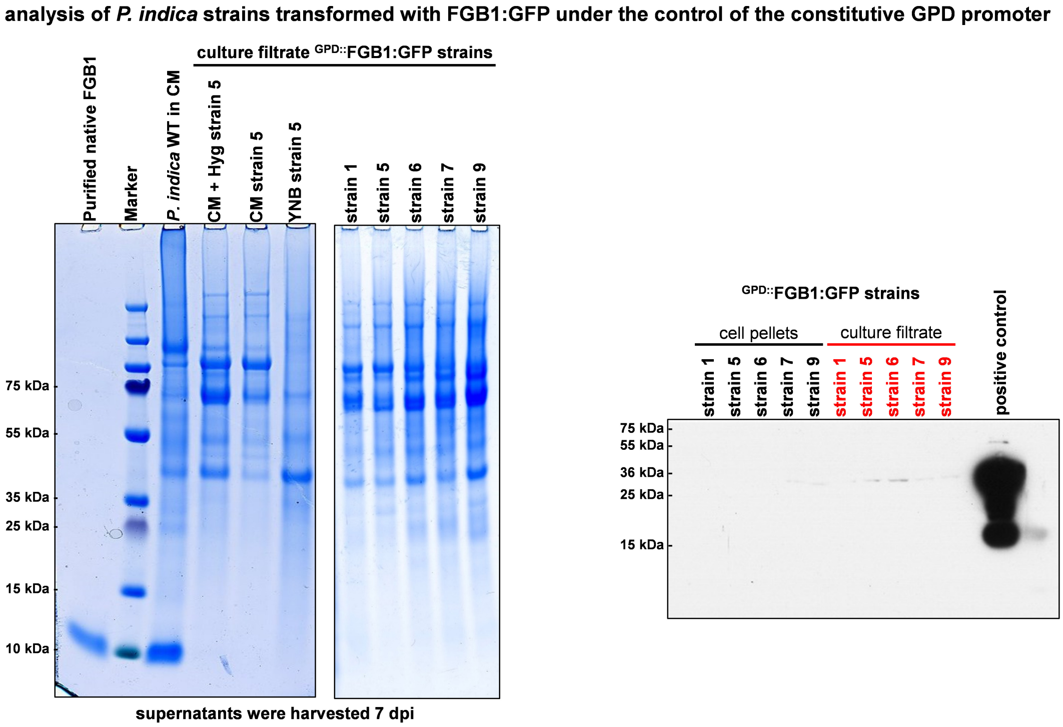

Supplementary Figure 5: *P. indica* strains transformed with FGB1:GFP under the control of the constitutive *Pi*GPD promoter are impaired in the production of native FGB1 and show hardly any protein production of the fusion construct. Left: CBB stained SDS-PAGE showing the secreted protein profiles of *P. indica* WT and strains tested positive for the ^{PromGPD}FGB1:GFP construct after 7 days of growth in CM-medium. All transformed clones analyzed showed no significant levels of native FGB1 in the secreted protein fractions compared to the wild type, possibly indicating an effect termed quelling related to co-suppression observed in plants and RNA interference in animals. Right: Anti-GFP western blot of supernatant and pellet protein preparations of the samples shown in the SDS-PAGE on the left.

Supplementary Figure 6:

a **FGB1:GFP localizes to the cell wall and is secreted by *U. maydis* SG200**

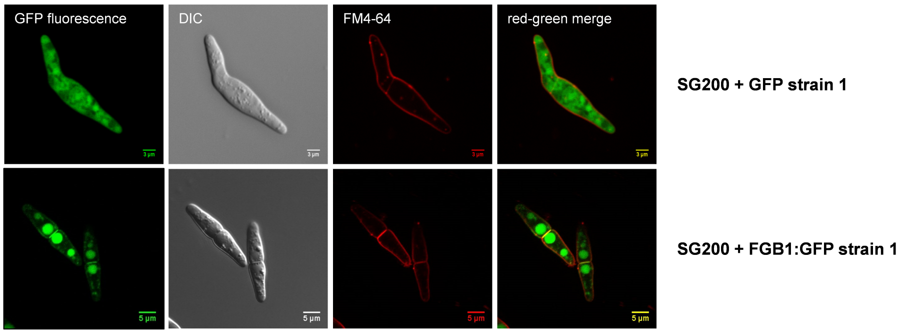

FGB1:GFP signals are retained on the cell wall of *U. maydis* SG200 after plasmolysis with 1.5 M NaCl

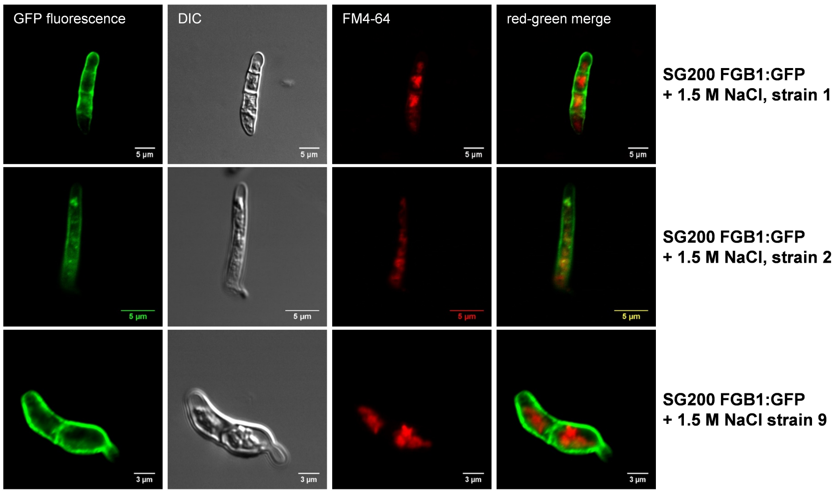

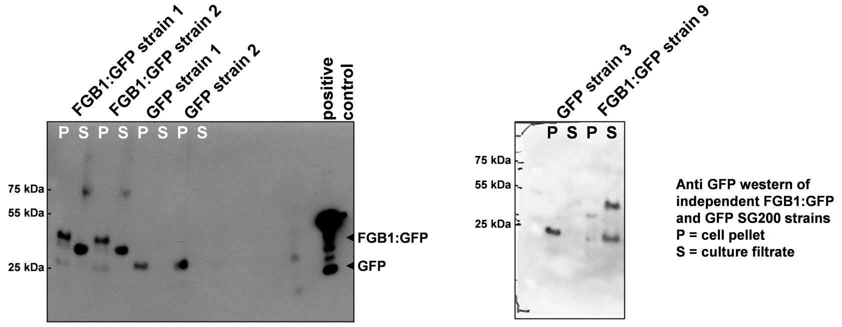

b **FGB1:GFP expression in *U. maydis* SG200 does not affect filamentation but confers resistance to Congo red**

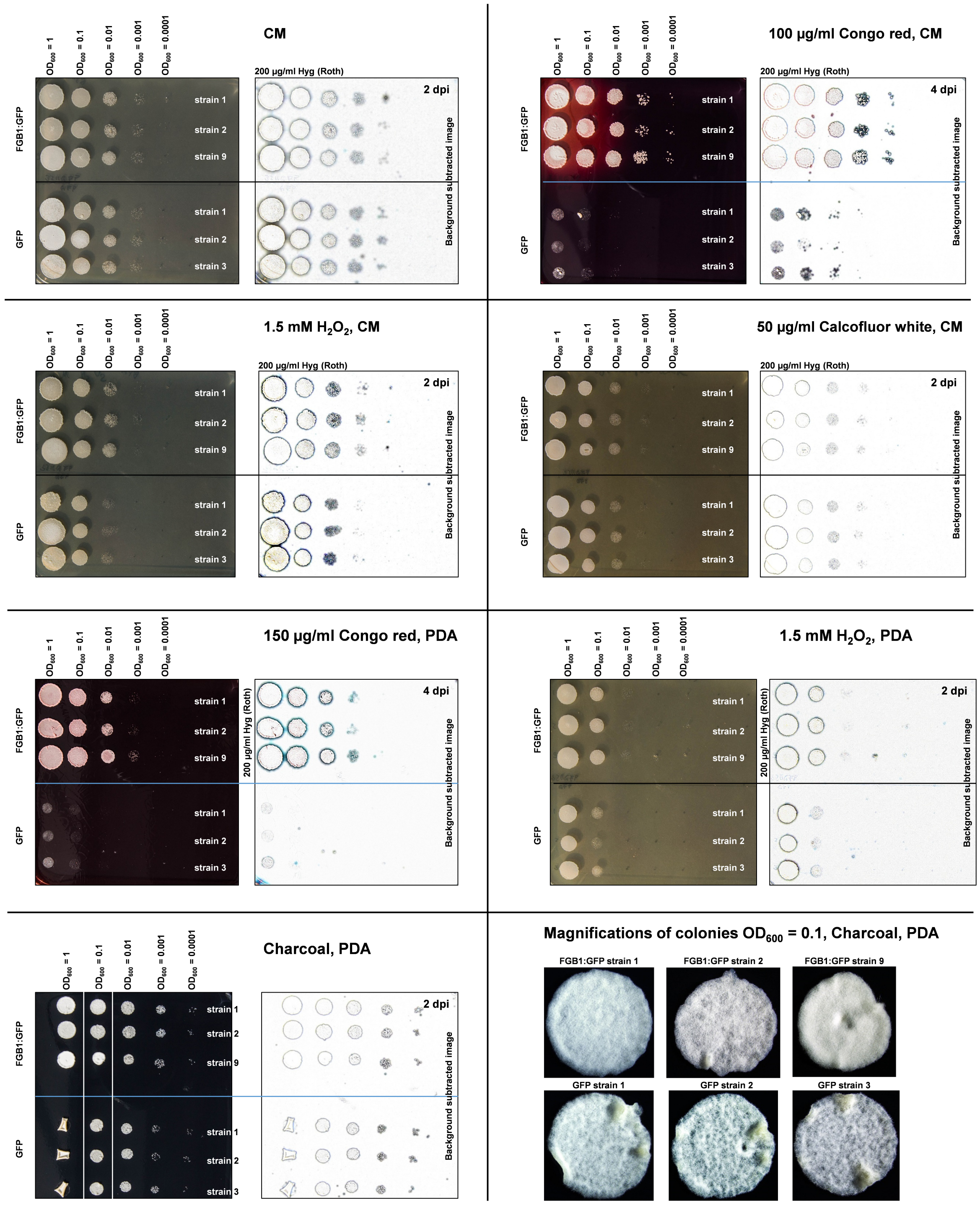

Supplementary Figure 6: a FGB1:GFP localizes to the vacuole and the outer border of *U. maydis* SG200 and is secreted into the culture medium. Green channel: confocal microscopy images showing the GFP signals of *U. maydis* SG200 cells either transformed with pGoGFP or pGoFGB1:GFP. The red channel shows the localization of the membrane stain FM4-64 after 5 min incubation at RT. DIC depicts differential interference contrast images. FGB1:GFP is present on the cell wall of the respective *U. maydis* transformants. After plasmolysis using 1.5 M NaCl the collapsed cell interior

showed FM4-64 staining while GFP signals remained at the cell wall. Developed western blot obtained after probing the cell pellets (P) and TCA precipitated supernatants (S) of pGoGFP SG200 or pGoFGB1:GFP SG200 strains grown in YPES-light with an anti-GFP antibody. **b** Expression of ^{PromGPD}FGB1:GFP in *U. maydis* confers increased resistance towards the cell wall stress agent Congo red. Drop dilution series of *U. maydis* SG200 shows the growth of the transformed strains on CM and potato dextrose agar (PDA) plates containing the indicated Congo red, Calcofluor white or H_2O_2 concentrations. No obvious difference in the filamentation behavior of the transformants was observed on PDA-charcoal. Magnified images show the colonies obtained for the drop dilution on the PDA charcoal plate with an inoculum of OD_{600} = 0.1. All plates contained 200 μg/ml hygromycin.

Supplementary Figure 7:

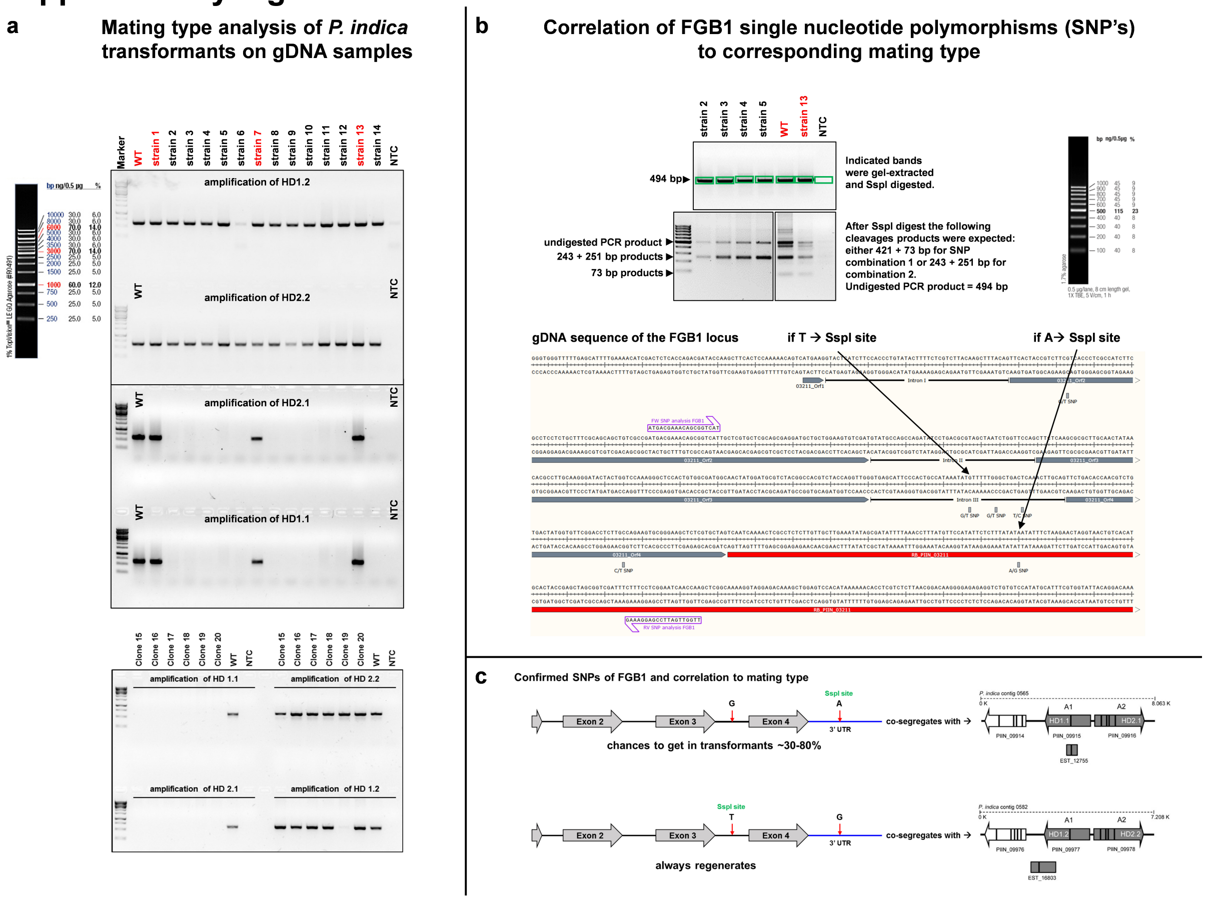

Supplementary Figure 7: About 40-85% of all regenerated clones obtained after transformation of wild type *P. indica* are homokaryotic. **a** The agarose gels show exemplarily for 20 clones the products obtained after analytical PCR probing for the *P. indica* mating type genes PIIN_09914/PIIN_09915 and PIIN_09976/PIIN_09977 that was performed on gDNA templates of regenerated clones after the attempt to delete FGB1 from the *P. indica* genome with a construct consisting of a hygromycin cassette flanked by ~1000 bp of the up- and downstream sequences of the FGB1 gene. In all cases the resistance cassette was randomly integrated into the genome. **b** shows the analysis of selected clones used to correlate specific single nucleotide polymorphisms (SNP's) in FGB1 to the corresponding mating type. Therefore, the indicated region of the FGB1 locus was amplified, gel purified, subjected to a limited SspI restriction digest and analyzed by gel electrophoresis. The results show that only one set of the possible FGB1 SNP combinations was present in the strains that were only positive for the PIIN_09976 and PIIN_09977 genes. **c** Schematic summary of the combined results shown in a and b. WT = *P. indica* wild type gDNA sample; NTC = non template control

Supplementary Figure 8:

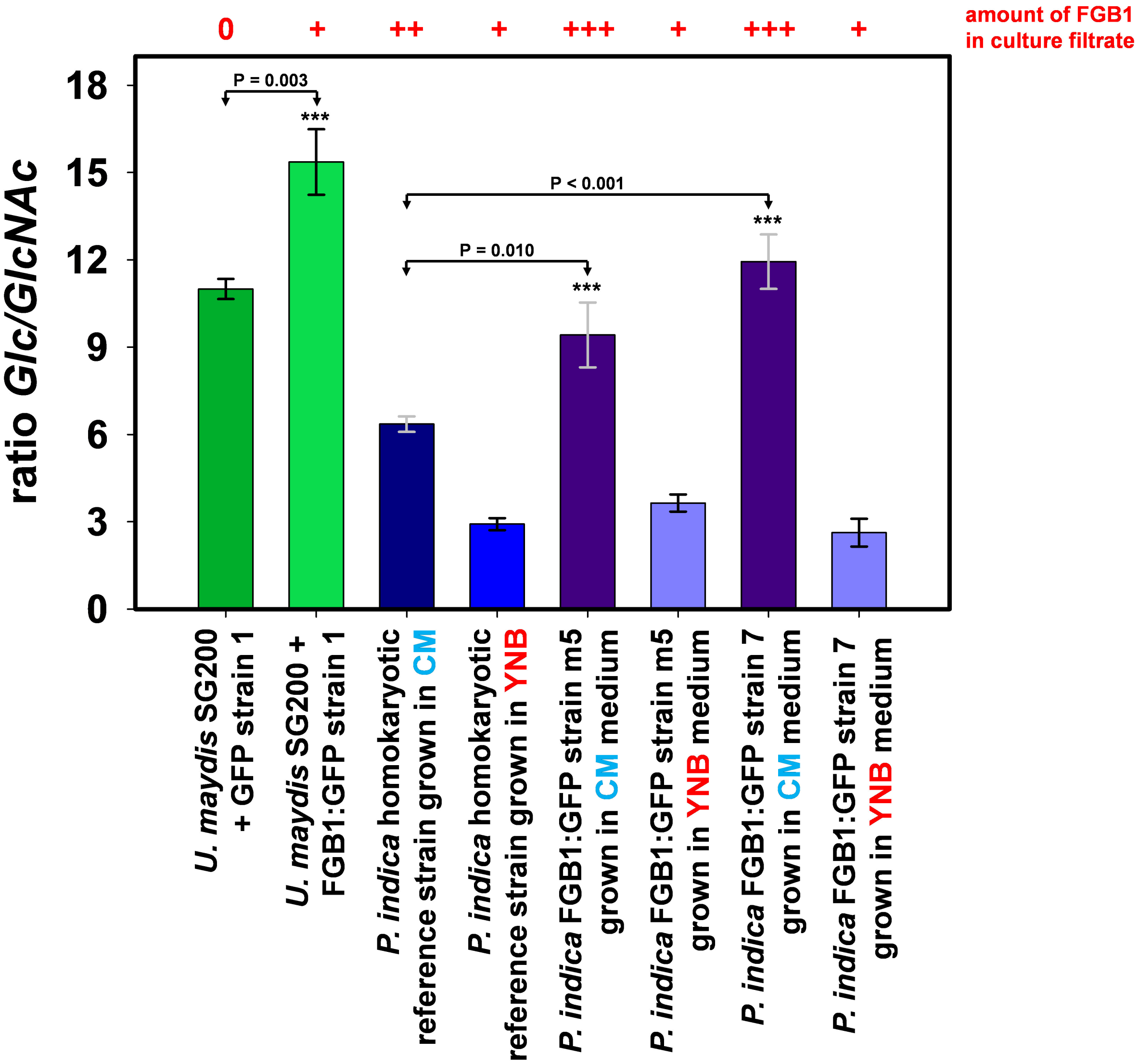

Supplementary Figure 8: Glucose to *N*-acetylglucosamine ratio determined by ^{1}H-NMR spectroscopy after total hydrolysis of protein and membrane free cell wall preparations. All experiments were performed in three independent biological replicates grown under identical conditions (28°C, 120 rpm shaking). *Ustilago maydis* was grown in YPES-light and *P. indica* strains in either CM or YNB media. Error bars show the standard deviation. Significance was calculated using the paired t-test algorithm of SigmaPlot (v11). 0, +, ++ and +++ indicate the relative amount of FGB1 present in the respective culture filtrates deduced from Coomassie blue stained SDS-PAGE gels and/or western blots.

Supplementary Figure 9:
FGB1 does not inhibit glucanase activity

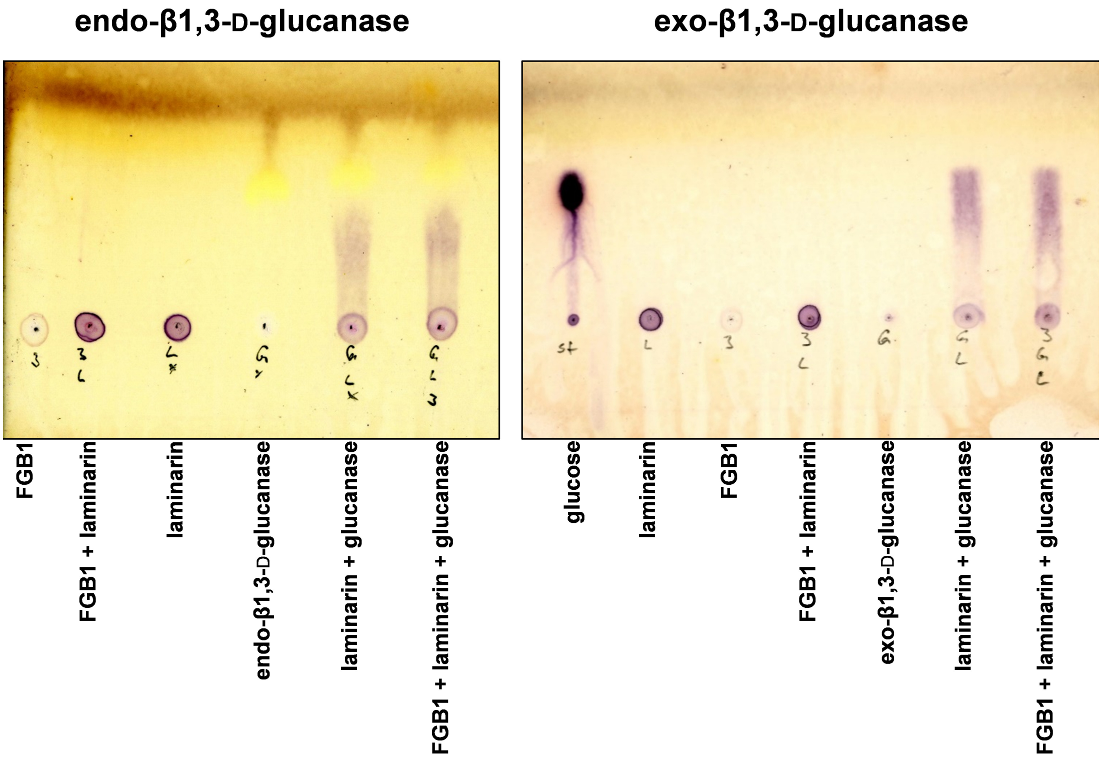

Final assay concentrations:
116 µM FGB1
48 µM laminarin
0.025 mg/ml endo-β1,3-D-glucanase
(*Hordeum vulgare* EC 3.2.1.39)

Final assay concentrations:
116 µM FGB1
48 µM laminarin
0.1 mg/ml exo-β1,3-D-glucanase
(*Trichoderma virens*)

Glucose liberated from laminarin by glucanase activity in presence and absence of FGB1

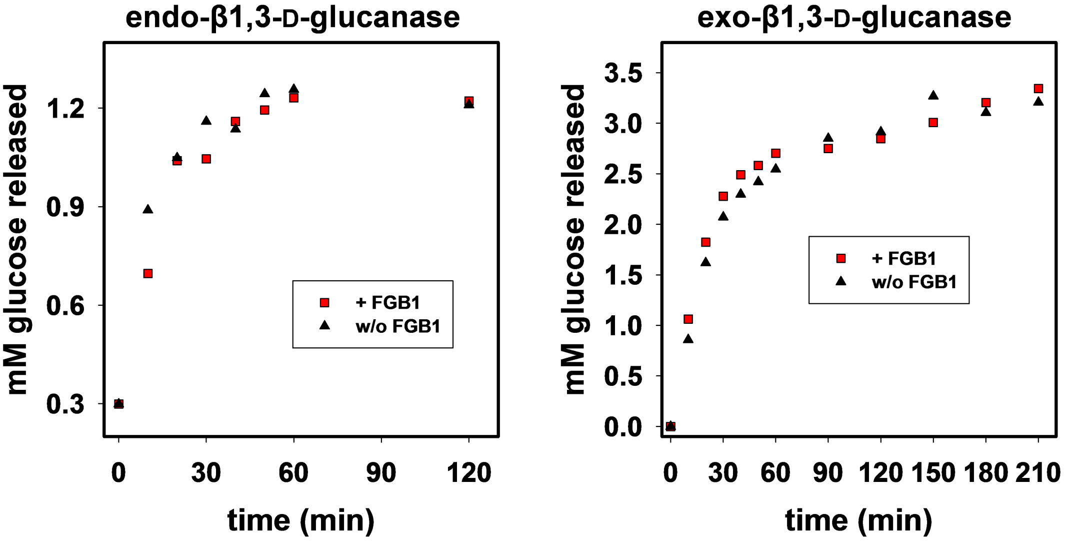

Supplementary Figure 9: Top: FGB1 does not protect laminarin from hydrolysis by endo-β1,3-D- and exo-β1,3-D-glucanase. Thin layer chromatography showing the laminarin hydrolysis of endo-β1,3-D- (left) and exo-β1,3-D-glucanase (right) in presence or absence of FGB1. In case of the endo-

glucanase the assays were incubated for 15 min at 37°C, for the exo-glucanase hydrolysis was carried out for 1 hr at 37°C. 6 µl reaction assay was spotted per sample. TLC was performed in *n*-butanol:isopropanol:water (3:12:4) using silica-gel 60 F254 plates (Merck). After the run plates were dried at RT and developed by spraying with 1-napthol (15 mg of napthol in 12.4 ml ethanol + 1 ml H_2O + 1.6 ml H_2SO_4) and subsequent development at 200°C.

Bottom: Kinetic measurements showing the amount of glucose released from laminarin in presence and absence of FGB1. Laminarin ± FGB1 was incubated with either endo-1,3-β-D-glucanase (Barley, Megazyme® E-LAMHV) or exo-1,3-β-D-glucanase (*Trichoderma virens*, Megazyme® E-EXBGTV) in 100 mM sodium acetate buffer pH 5.2 or in 100 mM sodium acetate buffer pH 4.5 (12,5 or 25 U/l) respectively. Samples were taken at different time intervals and the reaction was stopped by heating the samples for 5 minutes at 95°C. The released glucose was quantified using the bicinchoninic acid (BCA)-assay via absorption measurements at 540 nm with a 96well plate reader (TECAN Sunrise). Each plate contained several glucose standards for calibration. Experiments were repeated 2-5 times with different FGB1 to laminarin concentration ratios. In none of the experiments performed any inhibitory effect of FGB1 on the glucose released by the glucanase activities was observed.

Supplementary Figure 10:

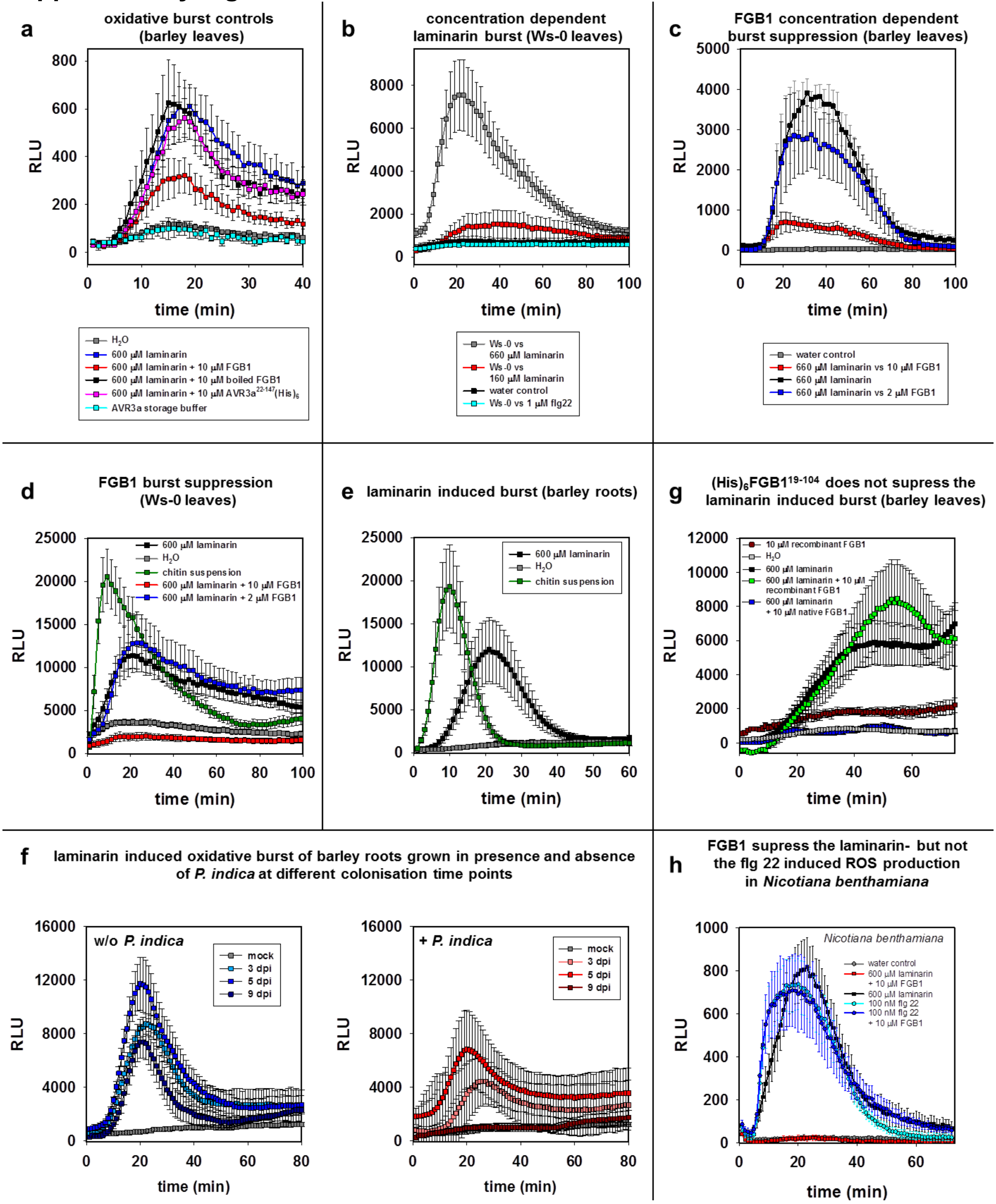

Supplementary Figure 10: FGB1 supresses the laminarin induced oxidative burst. **a** Barley leaf disks react with a strong ROS burst after stimulation with 600 µM complex laminarin (■) that was efficiently supressed with 10 µM FGB1 (■) but not with boiled FGB1 (■) or the unrelated *P. infestans* effector AVR3a (■). Neither the buffer control for AVR3a (■), the mock water control (■) not the buffer-laminarin control (■) affected ROS production. **b** Laminarin triggered ROS production in the *A. thaliana* ecotype Ws-0 is dependent on the concentration of the elicitor. ROS poduction after elicitation of leaf disks with 600 µM laminarin (■) is significantly higher then in the presence of 160 µM laminarin (■). No burst with 1 µM flg22 was observed (■). **c** Supression of the laminarin triggered oxidative burst by FGB1 is strongly dependent on the FGB1 concentration. Errors bars show the standard error of the mean of 6-8 technical replicates. **d** The laminarin triggered ROS production in the *A. thaliana* ecotype Ws-0 is suppressed in by FGB1. 600 µM laminarin (■) trigger ROS production which can be suppressed by 10µM of FGB1 (■) but not by 2µM of FGB1 (■). Chitin suspension, used

as a positive control triggered a ROS burst (■) whereas the mock water control did not (■). **e** Laminarin (■) and chitin (■) also elicit ROS production in roots of barley compared to the mock control water (■). Independent repetitions showed similar results. Error bars represent the standard error of the mean of 8 to 12 technical replicates. **f** Comparison of the oxidative burst after laminarin elicitation of barley roots either grown in presence of *P. indica* WT spores or after mock inoculation measured at the indicated time points. ROS production at the respective timepoints were measured on the same plate with the identical laminarin stock solution (600 µM final concentration) and were carried out in 8 technical replicates. For better visualization data are represented in two plots. **g** Native FGB1 but not (■) recombinant $(His)_6FGB1^{19\text{-}104}$ (■) is able to supress the laminarin triggered burst production of barley leaf disks (■). Recombinant FGB1 alone (■) or the water control (■) did not induce any ROS production. Error bars represent the standard error of the mean of 6 to 8 technical replicates. **h** The laminarin elicited (600 µM, ■) ROS production of *Nicotiana benthamiana* leaf disks can be sufficiently suppressed by 10 µM FGB1 (■). In contrast the flg22 (100 nM, ●) induced oxidative burst is not affected by the presence of 10 µM FGB1 (●).Error bars represent the standard error of the mean of 8 technical replicates.

Supplementary Figure 11:

U. maydis ^{PromGPD}FGB1:GFP strains are more virulent than ^{PromGPD}GFP control strains on maize

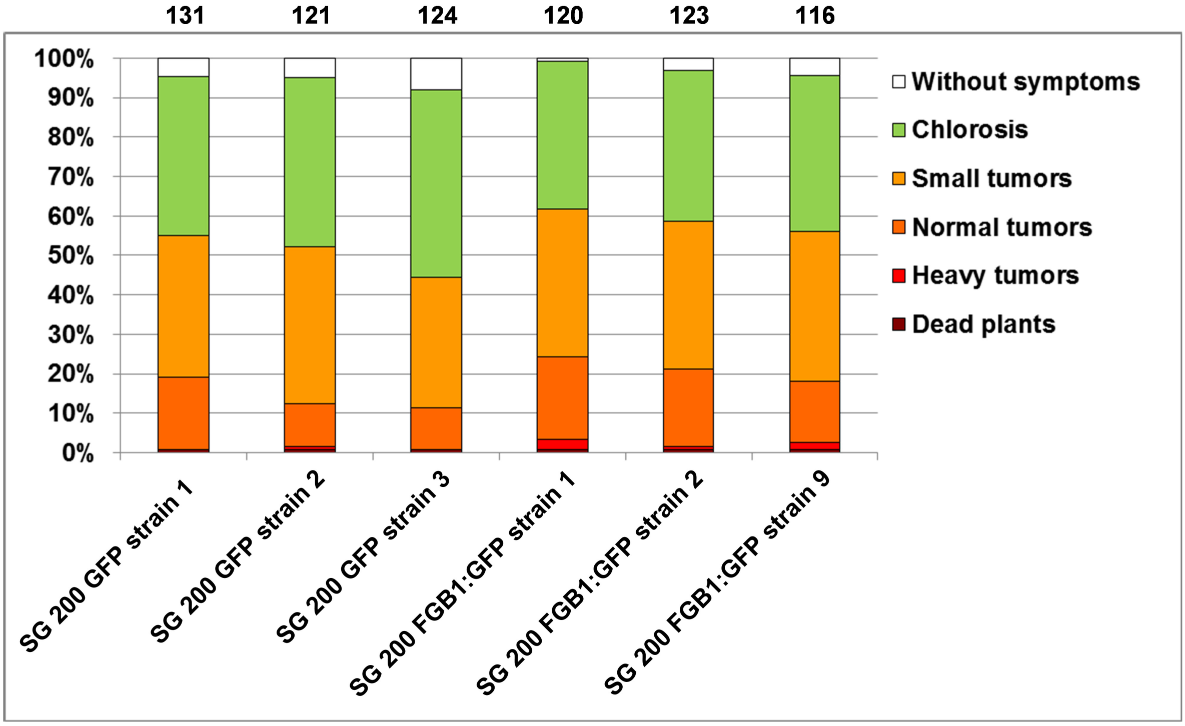

Supplementary Figure 11: Disease symptoms on maize plants were scored 12 days after infection with the indicated strains. Mean values of three independent infections are shown with the total number of infected plants indicated above each column. Disease scores are indicated on the right and were performed according to Djamei et al. (2011).

Supplementary Tables:

Supplementary Table 1: Main carbohydrate hydrolytic enzymes from barley detected in apoplastic fluid of *P. indica* colonized roots harvested at the indicated times.

Time	Accession	Description	Score	Coverage	# Unique Peptides	# Peptides	# AAs	MW [kDa]	Predicted Signal Peptide
**5 dpi**	none detected								
**10 dpi**	P00693	Alpha-amylase type A isozyme OS=Hordeum vulgare GN=AMY1.1 [AMY1_HORVU]	271.06	18.95	9	9	438	47.8	Yes 24|25
	**P34742**	**Glucan endo-1,3-beta-glucosidase GI OS=Hordeum vulgare PE=1 SV=2 - [E13A_HORVU]**	**199.06**	12.90	4	4	310	32.9	sequence not complete
	**P15737**	**Glucan endo-1,3-beta-glucosidase GII OS=Hordeum vulgare PE=1 SV=1 - [E13B_HORVU]**	**128.20**	14.67	5	5	334	35.2	Yes 28|29
	Q10NX8	Beta-galactosidase 6 OS=Oryza sativa subsp. japonica GN=Os03g0255100 [BGAL6_ORYSJ]	101.52	3.61	2	2	858	92.8	Yes 30|31
**14 dpi**	**Q02438**	**Glucan endo-1,3-beta-glucosidase GV OS=Hordeum vulgare PE=2 SV=2 - [E13E_HORVU]**	**296.90**	24.05	7	7	316	34.4	No
	**Q02126**	**Glucan endo-1,3-beta-glucosidase GIII OS=Hordeum vulgare PE=1 SV=1 - [E13C_HORVU]**	**261.78**	23.03	5	5	330	34.9	Yes 25|26
	**P34742**	**Glucan endo-1,3-beta-glucosidase GI OS=Hordeum vulgare PE=1 SV=2 - [E13A_HORVU]**	**259.82**	20.65	5	5	310	32.9	sequence not complete
	Q9FLG1	Beta-D-xylosidase 4 OS=Arabidopsis thaliana GN=BXL4 PE=1 SV=1 - [BXL4_ARATH]	245.69	4.46	4	4	784	84.3	Yes 38|39
	**P15737**	**Glucan endo-1,3-beta-glucosidase GII OS=Hordeum vulgare PE=1 SV=1 - [E13B_HORVU]**	**244.28**	18.26	5	5	334	35.2	Yes 28|29
	B6DZC8	Fructan 1-exohydrolase w3 OS=Triticum aestivum GN=1-FEHw3 [1FEH3_WHEAT]	222.03	14.09	8	8	596	66.3	Yes 20|21
	P00693	Alpha-amylase type A isozyme OS=Hordeum vulgare GN=AMY1.1 [AMY1_HORVU]	222.20	19.86	7	8	438	47.8	Yes 24|25
	Q7XCK6	Chitinase 8 OS=Oryza sativa subsp. japonica GN=Cht8 PE=2 SV=1 - [CHI8_ORYSJ]	204.56	7.28	2	2	261	27.5	Yes 29|30
	Q10NX8	Beta-galactosidase 6 OS=Oryza sativa subsp. japonica GN=Os03g0255100 [BGAL6_ORYSJ]	116.07	3.61	2	2	858	92.8	Yes 30|31
	Q9FXT4	Alpha-galactosidase OS=Oryza sativa subsp. japonica GN=Os10g0493600 [AGAL_ORYSJ]	107.46	5.04	2	2	417	45.8	Yes 55|56
	Q0INM3	Beta-galactosidase 15 OS=Oryza sativa subsp. japonica GN=Os12g0429200 [BGA15_ORYSJ]	52.20	1.96	2	2	919	100.9	Yes 31|32

Supplementary Table 2: Primer sequences

**Primers 5' to 3'**	**used for**
ATGACGAAACAGCGGTCAT	FW qPCR primer FGB1 (PIIN_03211)
TGAGAAGATCGACACTTCC	RV qPCR primer FGB1 (PIIN_03211)
GCAAGTTCTCCGAGCTCATC	FW qPCR primer *P. indica* TEF (PIIN_03008)
CCAAGTGGTGGGTACTCGTT	RV qPCR primer *P. indica* TEF (PIIN_03008)
ACTAATCGATATGAAGTTCACTACCGTCT	FW primer for amplification of FGB1 including the signal peptide from cDNA
CAGTAAGCTTGCACGAGAGCTTCCCGCAC	RV primer for amplification of FGB1 from cDNA lacking the stop codon
ATGACGAAACAGCGGTCAT	FW primer for FGB1 SNIP analysis
TTGGTTGATTCCGAGGAAAG	RV primer for FGB1 SNIP analysis
ATGAGTACGATTGCCCAAGG	FW primer for *P. indica* mating type locus HD2.1 (PIIN_09916)
TCGTCTCGTAGGCGACTTTT	RV primer for *P. indica* mating type locus HD2.1 (PIIN_09916)
CGATACCTACCCGCCTACAA<br>CTTTTTAAGCGGTGCTGGAG	FW primer for *P. indica* mating type locus HD1.1 (PIIN_09915)<br>RV primer for *P. indica* mating type locus HD1.1 (PIIN_09915)
ACATCTGGCTCCCATTTACG	FW primer for *P. indica* mating type locus HD2.2 (PIIN_09978)
GTTGAGCTTTGGCTCGTTTC	RV primer for *P. indica* mating type locus HD2.2 (PIIN_09978)
AGATATCCGGAGGCGAGTTT	FW primer for *P. indica* mating type locus HD1.2 (PIIN_09977)
CCTGAATCTGCTGTTCGTCA	RV primer for *P. indica* mating type locus HD1.2 (PIIN_09977)
tatacat.ATGcaccaccaccaccaccac<br>GCAGCAGCTGTCGCCGATGA	FW primer for cloning of FGB1 with N-terminal His6 tag into pET21b lacking the predicted signal peptide (*E. coli* expression)
TATAGAATTCCTAGCACGAGAGCTTCCCGCACT	RV primer for cloning of FGB1 into pET21b including stop codon
CAGTAGTGGCGGTCGAAGTG	qPCR-Primer_Barley (Ubiquitin gen) forward
ACCCTCGCCGACTACAACAT	qPCR-Primer_Barley (Ubiquitin gen) reverse
GGAGGGCGACAAGGTAAGTG	qPCR primer barley PR10 forward
CGTCCAGCCTCTCGTACTCT	qPCR primer barley PR10 reverse

DNA sequence of FGB1 showing the correctly annotated intron and exon segments that were identified through cDNA sequencing.

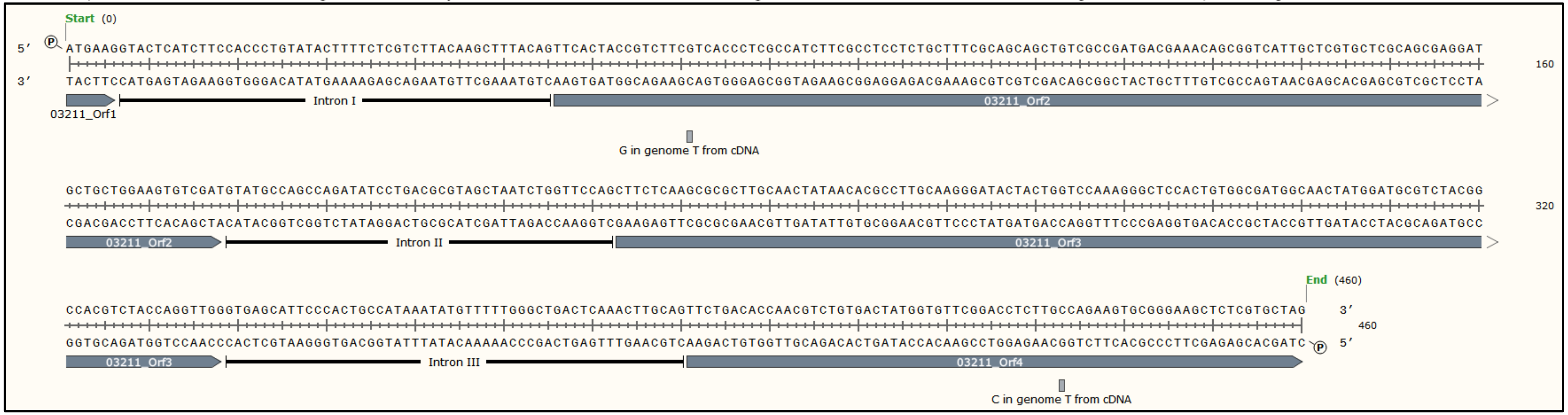

Translation to protein sequence:

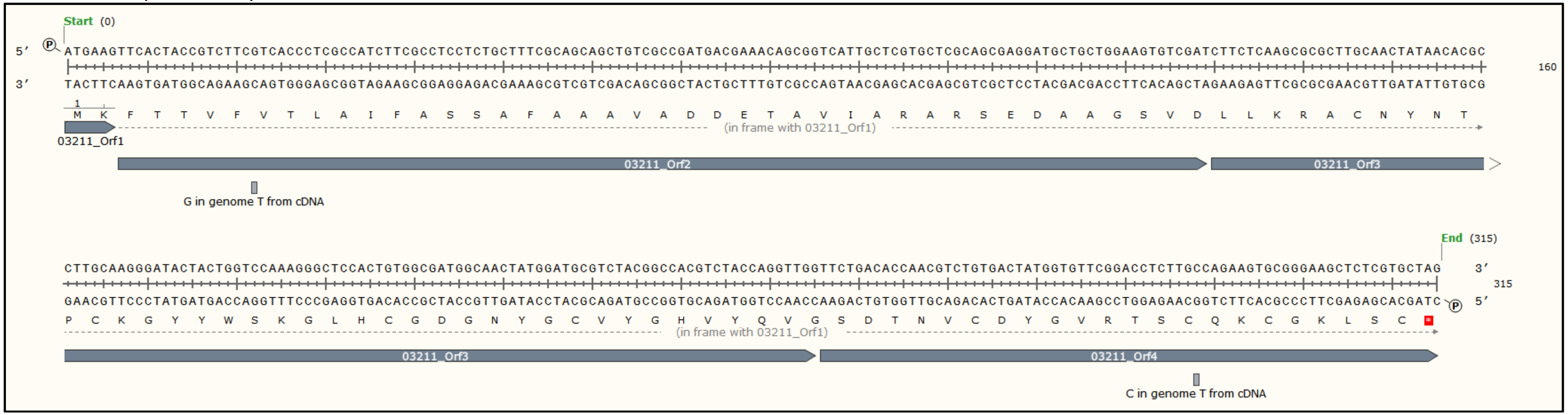

FGB1 promoter DNA sequence fragment obtained from GenScript

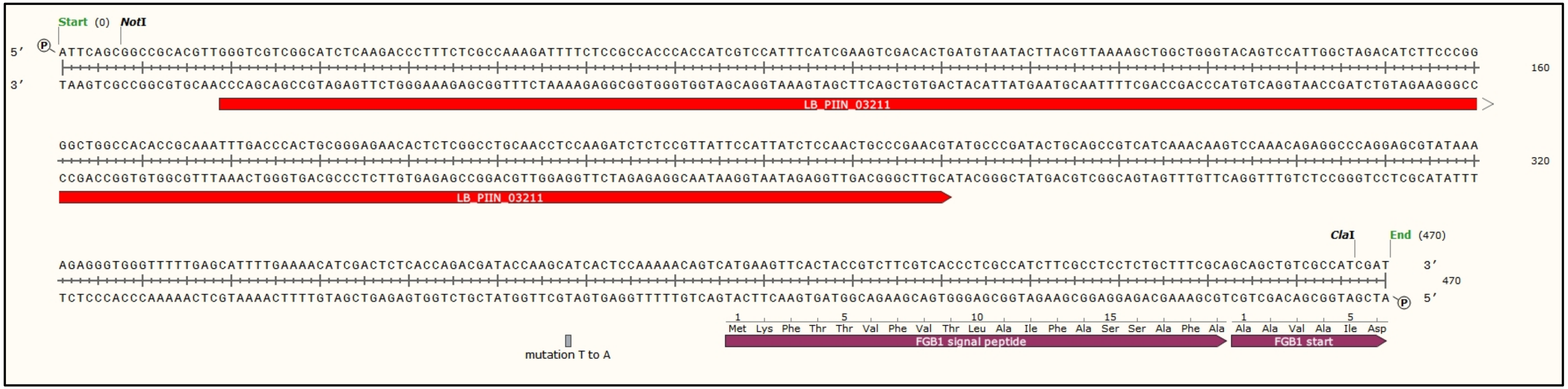

Supplement: Supplementary Information — Supplementary Figures 1-11 and Supplementary Tables 1 and 2 [file ncomms13188-s1.pdf]
